# General Anesthetics: Aspects of Chirality, Pharmacodynamics, and Pharmacokinetics

**DOI:** 10.3390/ph18020250

**Published:** 2025-02-13

**Authors:** Ružena Čižmáriková, Ladislav Habala, Jindra Valentová

**Affiliations:** Department of Chemical Theory of Drugs, Faculty of Pharmacy, Comenius University, Odbojárov 10, SK-833232 Bratislava, Slovakia; cizmarikovaruz@gmail.com (R.Č.); valentova@fpharm.uniba.sk (J.V.)

**Keywords:** general anesthetics, inhalational anesthetics, intravenous anesthetics, enantiomers, stereochemistry, chirality, pharmacodynamics, pharmacokinetics

## Abstract

The introduction of general anesthetics in the mid-19th century is considered one of the greatest contributions to medical practice. It was the first time that complicated surgical interventions became feasible, without putting an excessive strain on the patient. The first general anesthetics—diethyl ether, chloroform, and nitrous oxide—were limited by often severe adverse reactions and a narrow therapeutic window. They were later succeeded by modern anesthetics, with high anesthetic effect along with diminished toxicity. As with other medical drugs, many anesthetic compounds contain chiral centers in their molecules. Although currently used as racemates, the pharmacological activity of the respective enantiomerically pure antipodes can vary considerably, as can their adverse effects. Herein, we report on the available studies into the differences in bioactivity and toxicity between the enantiomers of chiral anesthetic agents. Both inhalational and intravenous anesthetics are discussed. Aspects of pharmacodynamics and pharmacokinetics are surveyed as well. The results could stimulate further research into the potential application of single-enantiomer anesthetics in clinical practice.

## 1. Introduction

General anesthetics are compounds that are mainly used in surgery to induce the state of general anesthesia, characterized by a loss of consciousness, a lack of response to painful stimuli, muscle relaxation, immobility, and suppressed undesirable somatic and autonomic nervous reflexes. They can be classified according to their mode of administration as inhalational and intravenous general anesthetics.

Many attempts at general anesthesia have been made since antiquity, using mainly herbal drugs, but the history of modern anesthesia starts in the 1840s when nitrous oxide and ether were first used to induce general anesthesia. Subsequently, a large number of new agents were introduced, yet only few of them became established in clinical practice in the long term [1].

Inhalational anesthetics enter the organism via inhalation in the lungs, from whence they are delivered by blood into the respective tissues. They mainly unfold their anesthetic effect in brain. They are gases or volatile liquids with high lipophilicity.

The ideal general anesthetic should exhibit a number of properties [2]:

- Easy to administer;
- Rapid onset as well as recovery from anesthesia;
- Easy and reliable maintenance of anesthesia in the desired stage;
- Sufficient analgetic effect;
- Muscle-relaxant properties;
- Broad therapeutic window (safety window);
- Minimum toxicity and adverse effects;
- Low degree of metabolism;
- Environmental safety;
- Low cost.

Inhalational anesthetics should additionally exhibit low blood–gas along with high oil–gas coefficients, be non-irritant to the respiratory tract, and be non-flammable. They include several chemically diverse types of compounds:

- Inorganic gases—nitric oxide, xenon;
- Hydrocarbons and non-halogenated ethers—cyclopropane, diethyl ether, ethylene, acetylene;
- Organohalogen compounds—chloroform, halothane, isoflurane, desflurane, sevoflurane, enflurane, methoxyflurane, chloroethane, trichloroethylene.

Many compounds that act as inhalational anesthetics are today only of historical importance, having been superseded by compounds with superb properties—higher efficacy and/or lower toxicity, as well as favorable pharmacokinetics. Several inhalational anesthetics formerly in use were abandoned because of their high flammability. Currently used agents include desflurane, isoflurane, sevoflurane, and nitrous oxide. Halothane is still used in some countries because of its affordability, whereas the use of xenon is still limited due to its high production costs. Anesthetics also find uses outside their main area of indication. Many of the inhalational anesthetics such as isoflurane and desflurane are used for severe epileptic seizures lasting for more than 5 min [2,3,4,5,6].

Another group of general anesthetics are intravenous anesthetics, which are often used in the initial phase of anesthesia [7]. In clinical medicine, especially in surgery, they are of paramount importance [8]. The history of modern i.v. anesthesia starts with the introduction of thiopental into clinical practice in 1934. Since then, many new compounds have been found to exert anesthetic effects. Despite their high efficiency and safety (in the induction of hypnosis, amnesia, analgesia, and immobility), their use is generally hampered by side-effects, especially cardiorespiratory depression. The most prevalent intravenous anesthetics include propofol, etomidate, ketamine, and barbiturates (such as thiopental). They tend to be combined with analgesics, especially opiates, as most of them do not produce this type of effect [9]. Propofol is the drug of choice for the induction of anesthesia, whereas etomidate is the most commonly used agent in patients with hemodynamic instability [10]. Thiopental is known to reduce intracranial pressure; hence, it is often used in cases of high intracranial pressure or head trauma [11]. It is also one of the most commonly used barbiturates in obstetrics because it penetrates the placenta very rapidly (within 30 s) [12]. Currently, thiopental is used as replacement therapy in critically ill patients with COVID-19 [13]. The anesthetic ketamine is a vital component of emergency medicine, offering a range of beneficial properties including analgesic, sympathomimetic, and dissociative effects [14].

In regard to their chemical structures, the intravenous anesthetics can be classified as follows:

- Barbiturate derivatives: thiopental, methohexital, hexobarbital;
- Phenolic derivatives: propofol, fospropofol;
- Steroid derivatives: alfaxalone/alfadolone;
- Derivatives of phenylacetic acid: propanidid;
- Various structures: ketamine, esketamine, etomidate.

The mechanism of action of central anesthetics has been the subject of much debate (e.g., [15,16,17,18]). Despite the fact that several theories have been proposed, there is still no final scientific agreement, and a detailed discussion is beyond the scope of this article.

Early on, it was hypothesized that the potency of volatile anesthetic agents (as described by the minimum alveolar concentration, MAC) correlates with their lipid solubility (the Meyer–Overton correlation). This theory attributes the effects at the cell level to a membrane-mediated mechanism, either directly changing the physicochemical properties of the lipid bilayer or modulating the activity of membrane proteins in the neuronal membrane. A quantitative expression of lipophilicity is the blood/gas partition coefficient, expressing solubility in blood. A substance that is more soluble in the blood has a slower distribution and a slower onset and cessation of action. Lipid solubility is expressed by the oil/gas partition coefficient; a more liposoluble anesthetic is more effective with and more readily distributed to adipose tissue, e.g., to the CNS. The physicochemical properties of selected inhalational anesthetics are given in Table 1 [4]. Later, the critical volume hypothesis (lipid bilayer expansion hypothesis) was proposed, stating that the accumulation of bulky and hydrophobic anesthetic molecules in lipophilic domains of the neuronal lipid membrane results in deformation and expansion due to a volume shift. Since then, more advanced variations of this hypothesis have been proposed, connecting the anesthetic effect to various perturbations of the lipid bilayer, such as thickness, elasticity, or lateral phase separation. A change in membrane properties may then result in the activation of an ion channel [19]. A recent hypothesis posits the non-specific binding of anesthetics to the binding site of the lipid palmitate, a lipid structure within the plasma membrane, in this way competing with the binding of cholesterol and in turn activating a potassium channel [19].

The lipid hypothesis is particularly attractive for the structurally simple and often chemically inert inhalational (volatile) anesthetics (which are chemically as different as sevoflurane, nitrous oxide, and xenon). The effect is considered to be non-specific, despite the structural diversity visible; the molecular volume of the anesthetic plays the major role, not its chemical structure. This was further corroborated by the observation that increases in ambient pressure reverse the anesthetic effect (pressure reversal effect) and that the anesthetic effects of inhalational anesthetics are cumulative (when using two different volatile anesthetics, the overall effect depends on the sum of their volumes).

Since the introduction of the lipid bilayer hypothesis, it has been challenged in several ways, e.g., the existence of enantiomers of chiral anesthetics differing considerably in their effects, despite having essentially identical physicochemical characteristics, including lipophilicity. Hence, hypotheses assuming direct binding to hydrophobic pockets of membrane proteins have been proposed. Thus, the GABA_A_ receptor was proposed as a major target of general anesthetics [20]. The neurotransmitter GABA_A_ (γ-aminobutyric acid or 4-aminobutyric acid) binds to certain receptors located on the cell membrane of pre- and postsynaptic neurons and opens ion channels, allowing the flow of negatively charged chloride anions into the cell and the flow of positively charged potassium cations out of the cell. This produces a change in membrane potential, causing hyperpolarization. This has been validated to a large extent for intravenous anesthetic agents such as etomidate or propofol.

Other (or concomitant) mechanisms of anesthetic activity have been proposed as well, e.g., the inhibition of glutamate-gated *N*-methyl-D-aspartate receptors (NMDA) or the activation of the membrane glycine receptor, which is part of the ion channel associated with the chloride channel of the plasma membrane [21,22]. Desflurane also activates the Ca^2+^-ATPase in the sarcoplasmic reticulum (SR), which transfers Ca^2+^ from the cytosol of the cell to the lumen of the SR at the expense of ATP hydrolysis during muscle relaxation. In addition, halothane and sevoflurane inhibit the nicotinic acetylcholine receptor and have an antagonistic effect on the *N*-methyl-D-aspartate receptor, which leads to the potentiation of the glycine receptor currents [23]. In sevoflurane, the inhibition of the 5-hydroxytryptamine subtype of the serotonin receptor has been reported [24].

For intravenous anesthetics, the mechanism of action is multireceptor in nature, similar to that of inhalational anesthetics, e.g., interaction with GABA_A_ receptors [25,26,27]. Nicotinic acetylcholine receptors (nAChRs) in the central nervous system may be a potential target for the anesthetic effects of thiopental [28,29]. Several studies have reported that anesthetics with antioxidant properties, such as propofol and thiopental, have a mechanism of action related to the inhibition of lipid peroxidation in tissue and the attenuation of reperfusion injury [30]. The selected properties of intravenous anesthetics mentioned in this article are given in Table 2 [4].

Given the large number of surgeries worldwide (estimated at 266 million in 2015), the demand for volatile anesthetics is significant, raising concerns about their environmental impact [31]. As most of them undergo minimal metabolism during clinical administration, they are mainly exhaled unchanged into the environment, leading to occupational exposure in operating theatres and post-anesthesia care units. Chronic occupational exposure to waste anesthetic gases can cause various adverse health effects in healthcare workers. Long-term exposure to halothane may cause mutagenesis and teratogenesis [32]. Isoflurane and sevoflurane have also been reported to cause dizziness, nausea, fatigue, headache, irritability, reduced mental performance, and cognitive decline [33,34].

On a global scale, nitrous oxide and fluorinated volatile anesthetics may contribute to the greenhouse effect and/or to the depletion of the ozone layer. The atmospheric lifetime of these gases varies from several years for halogenated anesthetics to more than a century for N_2_O. Global Warming Potential (GWP) is a measure of the environmental impact of volatile anesthetics in terms of CO_2_ equivalents; a greater GWP implies more heat captured per unit mass of CO_2_ and a greater contribution to global warming. One of the greatest contributors to GWP is the greenhouse gas desflurane, with a GWP of 3700 times that of CO_2_ [35]. In 1999, the global warming effect of volatile anesthetics was assessed to be 0.03% of the total effect [36]. Due to their widespread use and their long atmospheric half-lives, this contribution is likely to increase over time. The effect of inhaled anesthetics can be demonstrated using a simple analogy with the CO_2_ production during a car journey: the use of volatile anesthetics for 1 h at their typically employed concentrations is equivalent to driving 6.5 km for sevoflurane, 14 km for isoflurane, 95 km for nitrous oxide, and 320 km for desflurane [37,38]. Nitrous oxide, on the other hand, is recognized as one of the greatest contributors to ozone depletion [39]. Another anesthetic affecting the stratospheric ozone layer is isoflurane; this is due to its chlorine atom.

For these reasons, the reduction in anesthetic consumption and emissions has been a focus of research ever since. A number of different approaches have been considered, such as a switch from inhaled anesthetics to intravenous agents, avoiding N_2_O as a carrier gas, the application of recycling techniques for anesthetic gases (closed-circuit systems), or the use of xenon as a nonhazardous and environmentally friendly agent. To avoid occupational exposure in the operating room, a continuous fresh air ventilation system or a scavenger system must be used to prevent fume build-up and the connections within the anesthesia circuit must be carefully checked and adjusted [34,40].

The subsequent section proffers a compendium of selected pharmacological studies of common general anesthetics, either those currently employed or of greater historical significance. At present, due to pharmacological and economic considerations, the number of widely used general anesthetics is limited to a small number of agents. Selected pharmacodynamic and pharmacokinetic properties of the most commonly used inhalational and intravenous anesthetics are given in Table 3 [41].

## 2. Pharmacodynamics, Pharmacokinetics and Toxicity of Selected General Inhalational Anesthetics

### 2.1. Inorganic Gases—Nitrous Oxide (***1***), Xenon (***2***)

Among the inorganic gases, nitrous oxide (dinitrogen monoxide) maintains its place in modern anesthesia. Its advantage is its analgesic effect and effect on NMDA receptors, the suppression of wakefulness during anesthesia, and the possibility of its use also in at-risk patients with neurological or cardiovascular disease. In medicine, it is also used in a mixture with oxygen (85% N_2_O + 15% O_2_) as an anesthetic for short-term anesthesia. It is used for general anesthesia in surgery, gynecology, dentistry, and other outpatient procedures. The tests of its potential teratogenic effect in animal experiments and human studies have been inconclusive, although some association with increased reproductive loss might be indicated [42,43].

The 1:1 mixture of N_2_O and O_2_ known as ENTONOX is a safe and effective inhaled analgesic in situations where rapid onset and the reversal of effects are required. ENTONOX has an anxiolytic effect, i.e., it relieves patients from anxiety. Nitrous oxide is also frequently used in cryosurgery [44].

Xenon is also an effective anesthetic gas that has very suitable properties for medical use—zero metabolism, low blood gas ratio, a short recovery time, and hemodynamic stability. However, the large-scale use of xenon as a major anesthetic is severely limited due to its high cost of production (about 2000 times the cost of nitrous oxide), so it should only be applied in a completely closed circuit and its absorption and subsequent recycling should be ensured. Recently, there has been renewed interest in xenon as it has many of the properties of an ideal anesthetic [45,46].

### 2.2. Hydrocarbons and Non-Halogenated Ethers

Cyclopropane (**3**) and diethyl ether (**4**) are not used in clinical practice in most countries. The advantage of cyclopropane is the rapid onset of action and relatively rapid awakening. However, it is explosive, and its use is associated with a risk of cardiac arrhythmia [47,48]. It is evident that, in the absence of the potential hazards associated with its high degree of explosiveness and given the favorable hemodynamic profile and wide therapeutic index of cyclopropane (a property derived from the inherent chemical strain of its three-member carbon ring), there is a strong likelihood that this substance would have maintained its position within the domain of modern clinical practice.

Ethoxyethane (diethyl ether) has been in use for over 100 years. It is of low toxicity, has an analgesic and myorelaxant effect, and only slightly affects respiration and blood pressure. Because of its explosiveness, it has been gradually replaced by more modern anesthetics. Nevertheless, it was not until the 1960s that the role of ether as the standard general anesthetic was superseded by the increasing prevalence of fluorinated hydrocarbons. The agent still plays some role in underdeveloped countries due to its affordability. Moreover, the use of ether facilitates effective intra-operative pain management, which can persist for several hours into the postoperative period [49,50,51].

### 2.3. Halogenated Hydrocarbons and Ethers

A breakthrough in this group of anesthetics occurred with the introduction of fluorocarbons and fluorinated ethers into clinical practice [52]. As with other drugs, in addition to structure, physicochemical properties, and other factors, the stereochemical arrangement of these drugs plays a role in their biological activity [53,54].

**Figure 1:**
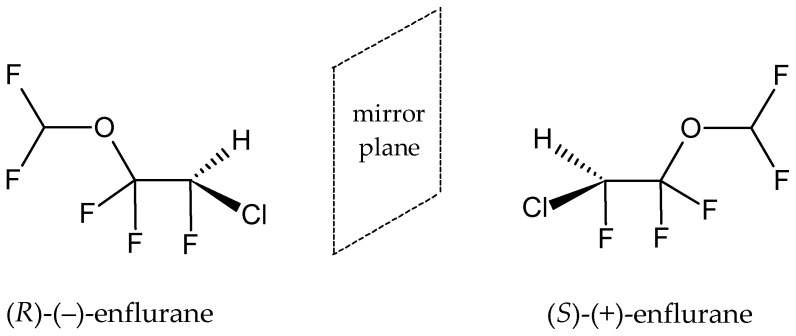
Enantiomers of enflurane (**7**).

In the structures of many anesthetics, especially in halogenated hydrocarbons and ethers, there is a stereogenic center on the carbon atom and the molecules are not identifiable with their mirror images and rotate the plane of polarizable light to the right (+) or to the left (−), leading to differences in their pharmacodynamic, pharmacokinetic, and toxicological properties. In terms of the Cahn–Ingold–Prelog system, the individual stereoisomers (+) and (−) have absolute configurations (*S*) and (*R*), respectively. An example is enflurane, whose dextrorotatory (+) isomer has an absolute configuration (*S*) and whose levorotatory (−) isomer has the configuration (*R*), as shown in Figure 1.

The group of inhalational anesthetics with a stereogenic center includes halothane (**5**), isoflurane (**6**), enflurane (**7**), desflurane (**8**), and 1,1,2-trifluoro-1-fluoromethoxy-2-chloroethane.

Halothane, IUPAC name: 2-bromo-2-chloro-1,1,1-trifluoroethane

**Figure 2:**
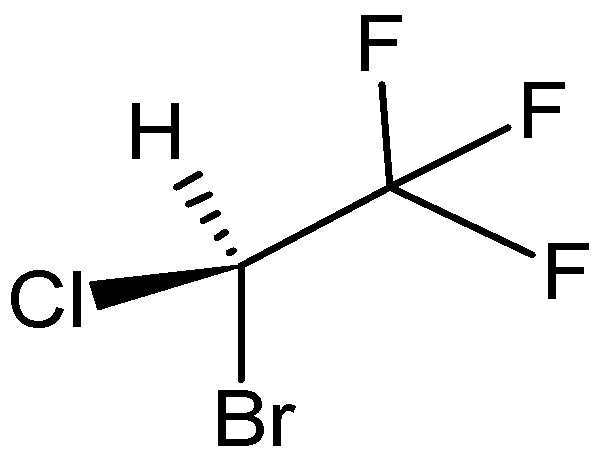
The structure of (*R*)-halothane (**5**).

Its structure classifies it as a halogenated hydrocarbon (Figure 2). It is a non-flammable, non-explosive, colorless liquid (boiling point 50.2 °C), and is stable in an alkaline environment. The moderate solubility of halothane in blood and its high narcotic activity allow the rapid onset of anesthesia (5 min from the start of inhalation of the anesthetic). It penetrates the CNS well using small doses of myorelaxants, and its muscle-relaxant effects are moderate. Although it is an effective anesthetic, it does not have a significant analgesic effect. Its instability is reduced by storage in dark bottles and the addition of 0.01% thymol. Halothane is a chiral molecule and is administered as a racemic mixture; there are few published data on the different effects of individual enantiomers. Experiments with *C. elegans* on the differential interaction of stereoisomers of halothane indicate the existence of a genetic pathway that controls sensitivity to halothane, and that both lipid and protein targets may mediate its effects [55]. A study demonstrated that the *R* isomer of halothane produces significantly higher amounts of TFA adducts than the *S* isomer. Hepatic toxicity is thought to be caused by the metabolization of halothane to trifluoroacetic acid. Approximately 20% of inhaled halothane is metabolized in the liver and the metabolites are excreted in the urine [56]. These drawbacks were the main reason for its replacement by novel halogen-containing anesthetics [57], albeit the degree of hepatotoxicity of halothane has been questioned recently [58].

Isoflurane, IUPAC name: (*RS*)-2-chloro-2-(difluoromethoxy)-1,1,1-trifluoro-ethane

**Figure 3:**
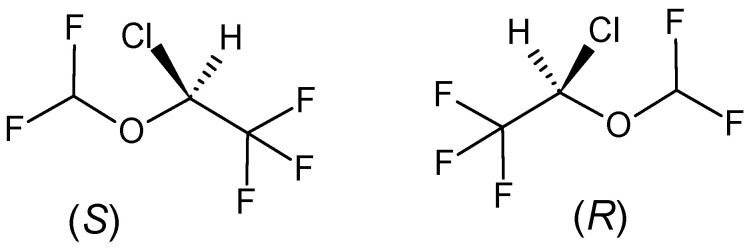
Isomers (*R*) and (*S*) of isoflurane (**6**).

It is a fluorinated volatile non-flammable anesthetic of the halogenated ether type (Figure 3). It is a colorless liquid with an ethereal odor and a boiling point of 48.5 °C. The solubility of isoflurane vapor in the blood and tissues of the human body is much lower than that of enflurane, which ensures faster the induction of anesthesia (2 to 3 min) and recovery from anesthesia (5 to 7 min). The effect of isoflurane on pulmonary artery vessels is similar to that of enflurane. However, there is more pronounced hypotension (due to the relaxation of the smooth muscles of blood vessels) and tachycardia with isoflurane anesthesia compared with all the above-mentioned anesthetics in this series. The low metabolism (0.17%) of isoflurane by cytochrome P450 and its low tissue solubility determine its lower nephro- and hepatotoxicity compared with previous anesthetics. Different pathways are favored for the metabolism of isoflurane by microsomes from the liver of rats and humans—human liver microsomes largely convert isoflurane into non-volatile fluorinated products, one of which appears to be trifluoroacetate. The predominant P450 isoform responsible for human clinical isoflurane metabolism in vivo seems to be P450 2E1 [59,60,61].

The first data describing pharmacological differences between the stereoisomers of the volatile anesthetic isoflurane, administered in vivo by the conventional route (inhalation), and by measurement of a clinically relevant anesthesia index, MAC, were published in a report [62]. Optical isomers of the inhalational general anesthetic isoflurane exhibited clear stereoselectivity in their effects on particularly sensitive ion channels in identified molluscan central nervous system neurons. At the median effective dose (ED50) for general anesthesia, the (+)-isomer was approximately twice as effective as the (−)-isomer in eliciting the anesthetic-activated potassium current *I_K(An)_* potassium current as well as in inhibiting the current mediated by neuronal nicotinic acetylcholine receptors. For the inhibition of the much less sensitive transient IA of potassium, the (−)-isomer was marginally more effective than the (+)-isomer. Both isomers were equally effective in disrupting lipid bilayers by their action on membrane proteins [63]. In [64], it was confirmed that this stereospecific effect of isoflurane on ion channels takes place directly on proteins. These findings lend further support to the hypothesis that the stereoselective effects on ion channels observed with isoflurane are attributable to direct actions on proteins rather than lipids.

Isoflurane also acts stereospecifically on γ-aminobutyric acid-type receptors [65,66,67], the (*S*)-(+) stereoisomer being the more effective inhibitor. The absolute potency differences between isomers are modest and measure-dependent. However, these effects are observed at clinically relevant concentrations of isoflurane. Furthermore, these effects are consistent with in vivo studies indicating that (+)-isoflurane is a more potent anesthetic than the (−)-isomer. On the other hand, there is no difference in binding to Ca^2+^ channels between the enantiomers compared to racemate [68]. This finding suggests that isoflurane, irrespective of its intracellular calcium levels, non-specifically inhibits the function of voltage-dependent calcium channels. This effect is believed to be facilitated by multiple binding sites.

The stereospecific binding of isoflurane to bovine serum albumin (BSA) was studied by ^19^F nuclear magnetic resonance, and stronger binding of the (*R*)-(−) enantiomer was observed in terms of dynamic binding parameters compared to the (*S*)-(+) stereoisomer [69].

Harris et al. [70] performed in vivo studies and administered intraperitoneal injections of isoflurane isomers to mice. The authors found a longer sleep duration with the (*S*)-(+) isomer. Similar evidence of the superior efficacy of the (*S*)-(+) isomer of isoflurane was demonstrated in a rat model where, by measuring a clinically relevant anesthesia index, the MAC (+) isomer was shown to be 53% more effective than the (−) isomer [62]. The opposite conclusion was reached by Eger et al. [71], who found no difference in minimum alveolar concentration (MAC) values for the two isoflurane enantiomers.

Enflurane, IUPAC name (*RS*)-2-chloro-1-(difluoromethoxy)-1,1,2-trifluoroethane

Enflurane (Figure 1) is a clear, colorless, sweet, volatile liquid that boils at 56.5 °C. Regarding its mechanism of action, it may increase the activity of the inhibitory neurotransmitter γ-aminobutyric acid on synaptic transmission and may also inhibit glutamatergic excitatory transmission [72]. As a very potent and stable general anesthetic, it was used to induce and maintain general anesthesia during surgery and caesarean section, and also for analgesia during vaginal delivery. Isolated cases of severe acute liver injury similar to halothane hepatitis have been observed and therefore it is currently being replaced by more modern volatile anesthetics.

A study of enflurane metabolism using cytochrome P450 2E1 from human livers confirmed that the metabolism of (*R*)-enflurane was significantly higher than that of (*S*)-enflurane (ratio 1.9:1), while the rate of racemic metabolism of enflurane was generally between the values observed for the (*R*)- and (*S*)-enantiomers [73]. It was demonstrated that the toxicity of the chiral fluorinated volatile anesthetics is closely related to their metabolism by hepatic cytochrome P450.

Desflurane, IUPAC name (*RS*)-2-(difluoromethoxy)-1,1,1,2-tetrafluoroethane

**Figure 4:**
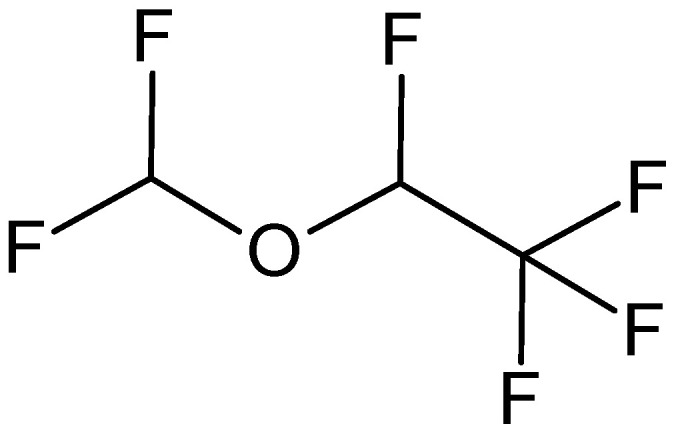
Chemical structure of desflurane (**8**).

It is chemically closely related to isoflurane, having a fluorine atom instead of a chlorine atom in its structure (Figure 4). Therefore, it has a much less harmful effect on the ozone layer. It is a non-flammable and non-explosive clear liquid with a pungent and irritating odor. It has the lowest boiling point of all volatile anesthetics (22.8 °C), and so a special vaporizer is required for its use. It is administered in the form of vapor, which is inhaled by the patient and induces deep, painless sleep (general anesthesia), which is maintained during the surgical procedure (operation).

Of all inhalational anesthetics, desflurane has the fastest onset and recovery from anesthesia and the most accessible regulation of responses. In adults, it is used to induce and maintain anesthesia; in children and infants, it is used only for its maintenance. It may cause tachycardia and respiratory irritation when administered at concentrations greater than 10% *v*/*v* [74,75]. Compared to other inhalation anesthetics, it has the weakest effect. Its disadvantage is also a higher price.

As with halothane, enflurane, and isoflurane, it is a racemic mixture of the optical isomers (*R*) and (*S*). Along with sevoflurane, it is gradually replacing isoflurane for human use, except in economically underdeveloped areas where its high cost precludes its use [76,77].

Among the non-chiral fluorinated inhalation anesthetics, methoxyflurane and sevoflurane are the best known.

Methoxyflurane, IUPAC name 2,2-dichloro-1,1-difluoro-1-methoxyethane

**Figure 5:**
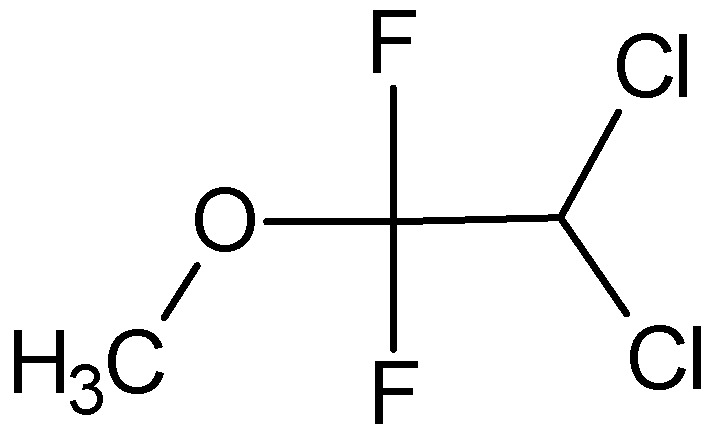
Chemical structure of methoxyflurane (**10**).

It is a halogenated ether (Figure 5) that was previously used clinically as a volatile inhalational anesthetic. Although methoxyflurane is known to be potentially nephrotoxic at anesthetic doses, the much lower doses used for pain relief are not associated with nephrotoxicity or an increased risk of kidney disease [78]. For this reason, for the past 30 years, it has been approved in many countries as an analgesic (in Australia, New Zealand, the United Kingdom, and Europe) for the emergency relief of moderate to severe traumatic pain. The analgesic use of methoxyflurane at subanesthetic doses in the Penthrox inhaler does not pose a risk of nephrotoxicity and, by comparison with tramadol in the treatment of acute musculoskeletal pain, was shown to be more effective and exhibited a more rapid onset of action in ankle injury [79].

Sevoflurane, IUPAC name 1,1,1,3,3,3-hexafluoro-2-(fluoromethoxy)propane

**Figure 6:**
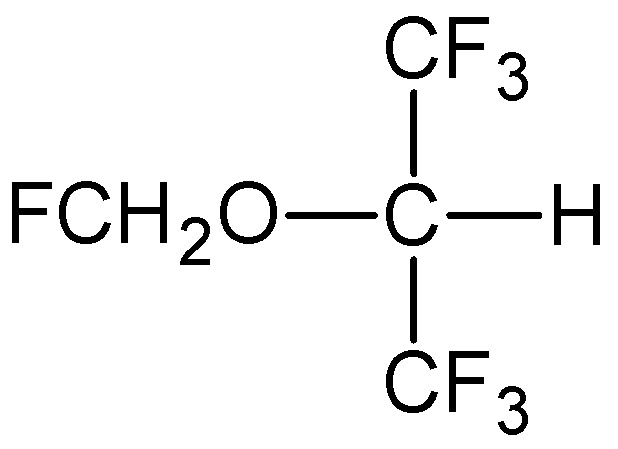
Chemical structure of sevoflurane (**9**).

Sevoflurane (Figure 6) is a colorless, transparent, inexplosive liquid with a boiling point of 58.5 °C. It is one of the most commonly used volatile anesthetics, especially for outpatient anesthesia in all age groups as well as in veterinary medicine [80,81,82]. It does not have good solubility in blood and tissues, which causes the onset of anesthesia 1–1.5 min after the beginning of the inhalation of the drug and is followed by the rapid cessation of anesthesia. Together with desflurane, sevoflurane replaced isoflurane and halothane in modern anesthesiology. It is often administered in a mixture of nitrous oxide and oxygen [83]. It has a less potent narcotic effect than isoflurane. Its effect on spontaneous respiration and airway smooth muscles of the bronchial tree is the same as that of desflurane. Sevoflurane is an inhaled anesthetic that is used frequently to induce and maintain anesthesia in children during surgery.

It is the only anesthetic that is not metabolized to trifluoroacetic acid and thus has a weak hepatotoxic effect. Less than 5% of absorbed sevoflurane is metabolized by cytochrome P450 2E1 to form hexafluoroisopropanol (HFIP) with the simultaneous release of inorganic fluoride and carbon dioxide (or a one-carbon fragment). HFIP is then rapidly conjugated with glucuronic acid and excreted as a urinary metabolite. No other metabolic pathways of sevoflurane have been identified [84]. Due to effects that are mediated by γ-aminobutyric acid type A (GABA_A_) receptors expressed in different cell types in the lung, preclinical data indicate that sevoflurane and other inhaled anesthetics attenuate pulmonary inflammation and dilate the airways. In an animal model of acute respiratory distress syndrome, sevoflurane has been shown to ameliorate the lung inflammatory response and enhance oxygenation to a greater extent than propofol [85]. It is currently recommended to add sevoflurane to the mixture used to treat COVID-19 patients on artificial ventilation [86].

## 3. Pharmacodynamics, Pharmacokinetics and Toxicity of Selected Intravenous Anesthetics

Thiopental, IUPAC name 5-ethyl-5-pentan-2-yl-2-sulfanylidene-1,3-diazinane-4,6-dione

**Figure 7:**
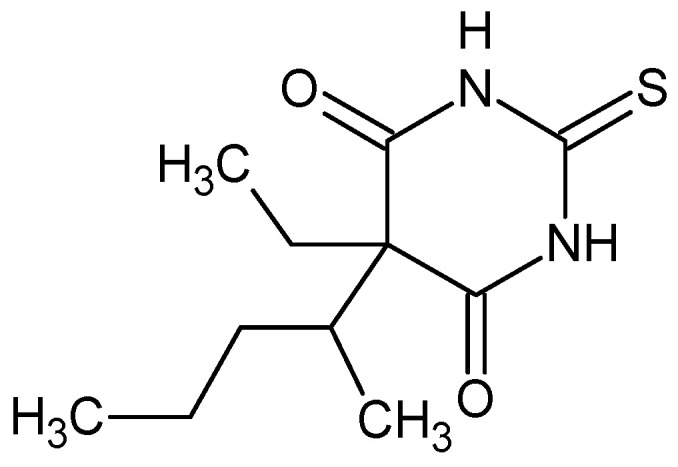
Chemical structure of thiopental (**11**).

Thiopental is a yellow-white hygroscopic powder with an odor of garlic and a melting point of 158–160 °C. It is a substituted derivative of 2-thiobarbituric acid with an ethyl group and a pentan-2-yl group at C-5 (Figure 7). Thiopental is prepared for clinical use as a sodium salt soluble in water and alcohol (a mixture of thiopental and sodium carbonate) [87,88]. The solutions are unstable and must therefore be prepared just before use and only applied intravenously, as they are irritating when used topically. It is an ultra-short-acting injectable anesthetic used at the onset of anesthesia. It induces hypnosis within 30 to 40 s after intravenous injection. This lipophilic molecule penetrates the blood–brain barrier well in its undissociated form (partition coefficient of about 10). Among its characteristic features are the transfer of thiopental from the CNS to less vascularized tissues, mainly adipose, and the persistence of a lower level of attenuation after the recovery of consciousness. It significantly dampens the respiratory center [89,90].

Thiopental has one stereogenic center in its structure and can thus occur in the form of two enantiomers, *R* and *S*. In clinical practice, it is used as a racemate. However, various papers have been published to compare the efficacy of both enantiomers [91,92]. The findings of a particular study indicated an inability to ascertain any pharmacokinetic disparities of clinical significance between the *R*-(+)- and *S*-(−)-thiopentone enantiomers and concluded that the observed minor differences could be explained by enantioselective differences in serum protein binding [93]. (*S*)-(−)-thiopental is approximately twice as active as (*R*)-(+)-thiopental in activating GABA_A_ receptors expressed in oocyte models from the African frog *Xenopus laevis*. This is consistent with the differential CNS depressant effect found in vivo [27,94]. Regarding chirality, the (*S*)-(−)-enantiomer is approximately 2-fold more narcotically potent than the (*R*)-(+)-isomer when tested in animals. The *S*-(−) isomer is the most potent anesthetic agent, followed by the *R*-(+) and the racemate. In terms of their capacity to impede pentylenetetrazol- and strychnine-induced seizures, the compounds are positioned in the following sequence of diminished potency: *S*-(−), racemate, and *R*-(+) [95,96].

As a highly lipophilic substance, thiopental binds to plasma proteins (about 70%) and is almost completely metabolized in the liver. Only 1% of the substance has been found to be excreted unchanged in the urine. It is administered intravenously as a 2.5–5% freshly prepared solution. After accounting for stereoselective binding, (*R*)-thiopental showed a 24% greater unbound fraction compared to (*S*)-thiopental, while no differences were found between the enantiomers in terms of unbound clearance and unbound steady-state volume of distribution [97]. In more recent work by Mather [98] in rats, total and unbound plasma concentrations of (*S*)-thiopental were found to be approximately 10–20% higher than those of (*R*)-thiopental, corresponding to higher clearance. Concentrations of (*S*)-thiopental in CNS tissue were approximately 20% higher compared with (*R*)-thiopental.

The main metabolite of thiopental is pentobarbital-(5-ethyl-5-(1-methylbutyl)barbituric acid, which also has two enantiomeric forms. The (*S*)-enantiomer causes a longer duration of sedation in patients than the (*R*)-enantiomer, and the clearance of the (*S*)-enantiomer is 25% lower than the clearance of the (*R*)-enantiomer [99,100,101].

**Figure 8:**
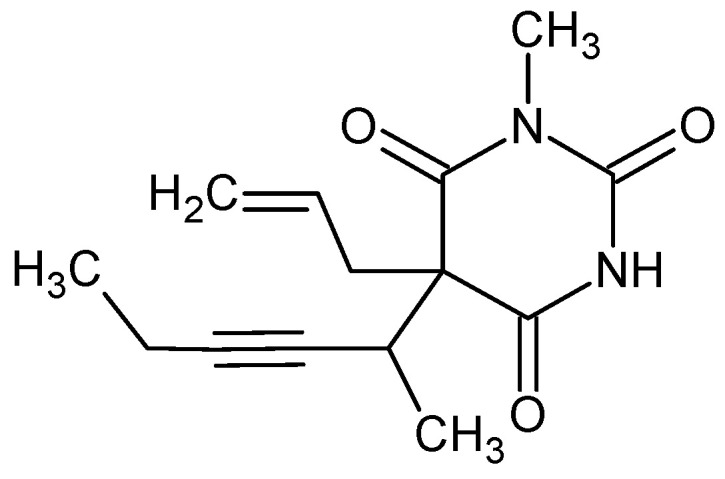
Chemical structure of methohexital (**12**).

Methohexital, IUPAC name 5-hex-3-yn-2-yl-1-methyl-5-prop-2-enyl-1,3-diazinane-2,4,6-trione (Figure 8)

It is a solid with a melting point of 96 °C (ethanol), a density of 1.113 g/cm^3^, and a pKa (predicted) of 7.82 ± 0.10 [93,102,103,104,105].

Methohexital is an ultra-short-acting methylated barbiturate with 2.5 times the hypnotic effect of thiopental. Methohexital binds to the chloride ionophore site of the γ-aminobutyric acid (GABA_A_)–chloride ionophore receptor complex, thereby enhancing the inhibitory effects of the GABA_A_ receptor in the brain. The result is synaptic inhibition, a reduction in neuronal excitability, and the induction of anesthesia. These experiments furnish evidence indicative of a functional coupling among GABA- and barbiturate receptors and the chloride ionophore. The evidence suggests that the GABA-activated chloride channel is a site of action for both agents—anesthetic and convulsant barbiturates. In addition, methohexital reduces glutamate (Glu) reactions [106,107,108].

Methohexital is interesting from a chirality point of view because it contains two stereogenic centers, one at position 5 in the barbiturate ring and the other at the first carbon of the 1-methyl-2-pentynyl side chain. The result is the formation of two pairs of stereoisomers. One pair are enantiomers and the other pair are diastereomers (i.e., they are not mirror images). Clinically, it is used as a mixture of the two least excitatory isomers. Metohexital and thiopental also show tautomerism (dynamic isomerism). In alkaline solutions, sodium thiopental is highly soluble in water due to the ionized side chain. When injected into plasma at pH 7.4, it takes on a non-ionized form that rapidly isomerizes to its tautomer, a highly fat-soluble compound [102,109,110]. Metabolism takes place in the liver via demethylation and oxidation [111].

**Figure 9:**
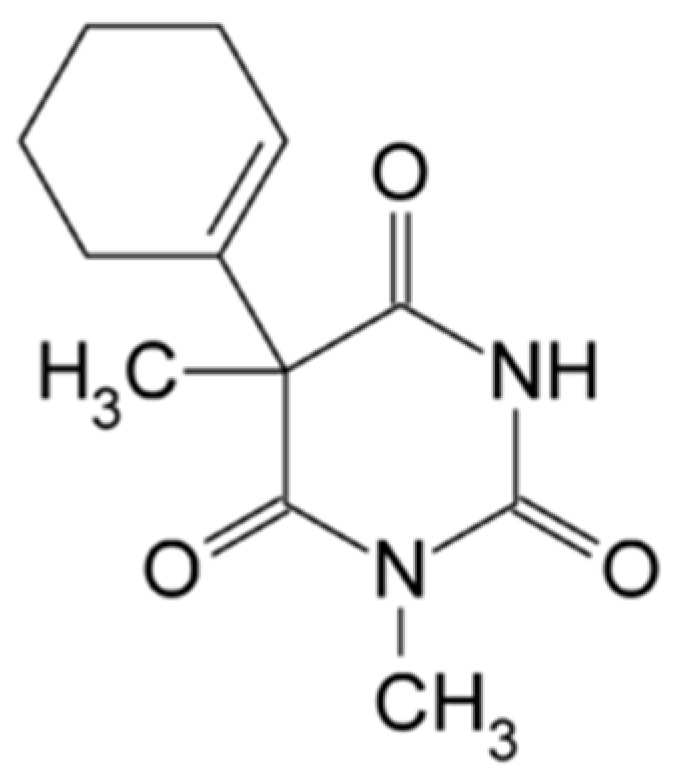
Chemical structure of hexobarbital (**13**).

Hexobarbital, IUPAC name 5-(cyclohex-1-en-1-yl)-1,5-dimethylpyrimidine-2,4,6(1*H*,3*H*,5*H*)-trione (Figure 9)

Hexobarbital is a racemic white powder with a bitter taste. It melts at 146.5 °C and has a dissociation constant of 8.2. Hexobarbital (also known as hexobarbitone) occurs in both an acid form and as a sodium salt and has hypnotic and sedative effects. It was used in the 1940s and 1950s as an effective means of inducing anesthesia for surgical procedures and as a fast-acting short-term hypnotic for general use since it has a relatively rapid onset and a short duration of action. Modern barbiturates (such as thiopental) have largely replaced the use of hexobarbital as an anesthetic because they allow better control of the depth of anesthesia [112,113]. Some scientific studies still use hexobarbital, for instance, a study focusing on the relationship between monoamine oxidase (MAO) activity and the hepatic content of cytochrome P450 (CYP), where the hexobarbital sleep test was used [114].

A higher in vivo narcotic potency of the (*S*)-(+)-isomer of hexobarbital has been shown to be associated with higher levels in the CNS compared with the (*R*)-(−) enantiomer. This appears to be due to an improved ability to pass through the blood–brain barrier. In an experiment on rats, on average, 43 mg/kg of (+)-hexobarbital was needed to achieve the effect, as compared with more than 114 mg/kg of (−)-hexobarbital. Approximately one quarter of the activity in the racemate was due to (−)-hexobarbital [115]. Hexobarbital as a short-acting hypnotic is metabolized to 3’-hydroxyhexobarbital by cytochrome P450 and then to 3’-oxohexobarbital by hepatic cytosolic dehydrogenase [116].

Saito et al. investigated the enzymatic function of recombinant CYP2C19 in enantiomeric hexobarbital (HB) 3’-hydroxylation and the role of amino acid residues such as Phe-100, Phe-114, Asp-293, Glu-300, and Phe-476 in CYP2C19 in stereoselective HB 3’-hydroxylation using a yeast cell expression system and a targeted mutagenesis approach [117]. The results indicated that Glu-300 and Phe-476 are important in the stereoselective oxidation of HB-enantiomers by CYP2C19.

Etomidate, IUPAC name ethyl 3-[(1*R*)-1-phenylethyl]imidazole-5-carboxylate

**Figure 10:**
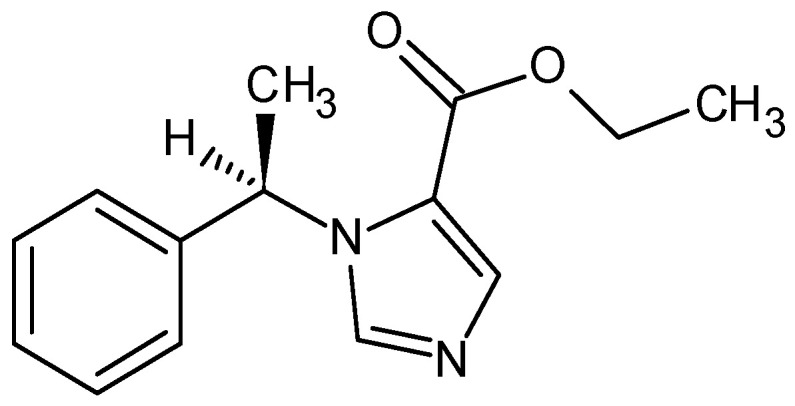
Chemical structure of etomidate (**14**).

Etomidate is a white powdery solid with a melting point of 72–74 °C and a boiling point of 160–162 °C (1 Torr pressure). It has a pKa of 4.24 (H_2_O, t = 25.0 °C) [118,119]. It is a non-barbiturate hypnotic with no analgesic effect (Figure 10). Its therapeutic index is 4 times greater than that of thiopental [120,121].

It was originally selected from a group of compounds that had been studied as anti-fungals and was found to be a potent hypnotic in animal studies. It was later found to be safer than the barbiturates which were in use. In clinical practice, it is a short-acting, intravenous, infiltrative, non-barbiturate-type anesthetic with an imidazole ring and a chiral center in its structure, and it is used to induce general anesthesia and sedation for short procedures and is also used in electroconvulsive therapy. In anesthesia, it has a wide therapeutic window, a rapid onset of action, and a safe cardiovascular risk profile. It is the only anesthetic that can reduce intracranial pressure and maintain normal arterial pressure. The duration of anesthesia is no longer than 10 min [122,123,124,125].

The (*R*)-(+) isomer was much more effective in potentiating GABA-induced currents, although the degree of stereoselectivity varied with anesthetic concentration. Both isomers were equally effective in disrupting lipid bilayers. These findings are consistent with the hypothesis that the primary mechanism of etomidate’s anesthetic activity is the modulation of the GABA_A_ receptor [126,127].

In clinical practice, it is used as a single isomer. The anesthetic effect lies primarily in the (*R*)-(+) enantiomer, which is approximately 5 times more potent than the (*S*)-(−)-isomer [116]. In individuals exhibiting no underlying pathology, the binding affinity of etomidate to proteins is estimated to be approximately 75%. It is also characterized by a large central volume of distribution (4.5 L/kg) and a large peripheral volume of distribution (74.9 L/kg), due to its high fat solubility [128,129]. The metabolism of etomidate in laboratory animals and humans depends on the activity of hepatic esterase, which hydrolyzes the drug to an inactive carboxylic acid and an ethanol leaving group [125,130].

**Figure 11:**
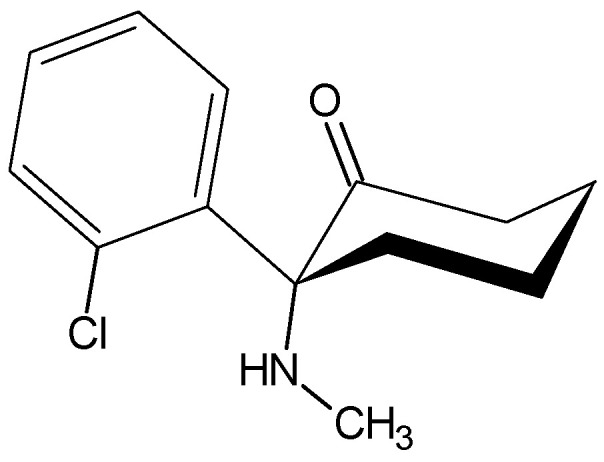
Chemical structure of ketamine (**15**).

Ketamine, IUPAC name (*RS*)-2-(2-chlorophenyl)-2-(methylamino)cyclohexanone (Figure 11)

Ketamine occurs as a free base with a melting point of 92–93 °C. It forms with hydrochloric acid a salt in the form of a white powder, is soluble in water, methanol, and ethanol, and is slightly soluble in chloroform. It is available in ampoules and dark glass bottles in the form of a clear, colorless solution at a concentration of 1%, 5%, or 10%. This is intended for direct use [131,132,133,134]. Ketamine is a relatively short-acting anesthetic with moderate analgesic activity. A characteristic feature of ketamine is its capacity to suppress the functionality of specific regions of the central nervous system while simultaneously enhancing the activity of others, thereby precipitating a state of so-called dissociative anesthesia [135,136]. In addition to its use in the human field, it also has applications in veterinary anesthesia [137,138,139,140,141].

In addition to its primary effects, ketamine has been demonstrated to elicit a number of other pharmacological responses. The use of this medication has been described in the treatment of chronic and neuropathic pain [142,143,144,145,146,147], as an antidepressant [148,149,150], in the management of psychiatric disorders [151,152], to facilitate the withdrawal from alcohol and heroin dependence [153,154], in the management of epilepsy [155,156], and in palliative care [157,158].

The primary site of metabolization is the liver, where cytochrome P450 is responsible for the conversion of the parent compound into its major metabolite, norketamine. The biotransformation of ketamine corresponds to *N*-dealkylation, the hydroxylation of the cyclohexane ring, conjugation to glucuronic acid, and the dehydration of hydroxyl metabolites to form cyclohexane derivatives. Experiments were conducted on the binding of the racemic form and both enantiomers of ketamine to α_1_-acid glycoprotein; no differences in binding were found. [159,160,161,162,163,164].

**Figure 12:**
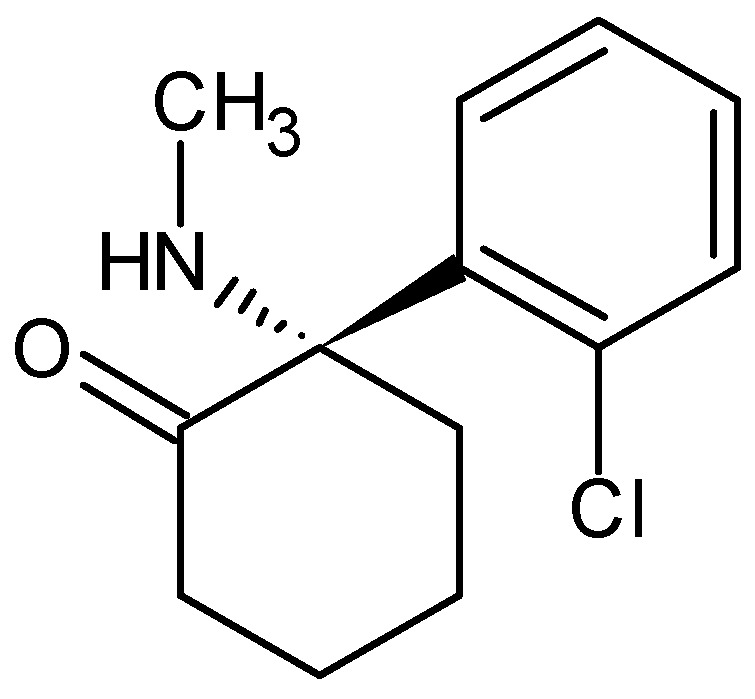
Chemical structure of esketamine (**16**).

Esketamine, IUPAC name (2*S*)-2-(2-chlorophenyl)-2-(methylamino)cyclohexan-1-one (Figure 12)

Esketamine is the dextrorotatory isomer of ketamine, which is approximately 2 times more potent as an anesthetic compared to racemic ketamine. It is excreted from the body more rapidly than arketamine ((*R*)-(−)-ketamine) or racemic ketamine, although arketamine slows its excretion [165,166,167].

Esketamine is 8 times more potent at inhibiting dopamine transporters than arketamine, thereby increasing dopamine activity in the brain. In contrast, ketamine exhibits no stereoselectivity for norepinephrine and serotonin transporters. At doses that produce the same intensity of effect, esketamine is generally considered to be more tolerable by patients [168].

Like ketamine, esketamine is a fast-acting antidepressant [169]. In 2019, it was approved in the United States for use with other antidepressants for the treatment of depression in adults, provided it is administered in a clinical setting [170]. In August 2020, it was approved by the U.S. Food and Drug Administration (FDA) for the short-term treatment of suicidal ideation [171].

**Figure 13:**
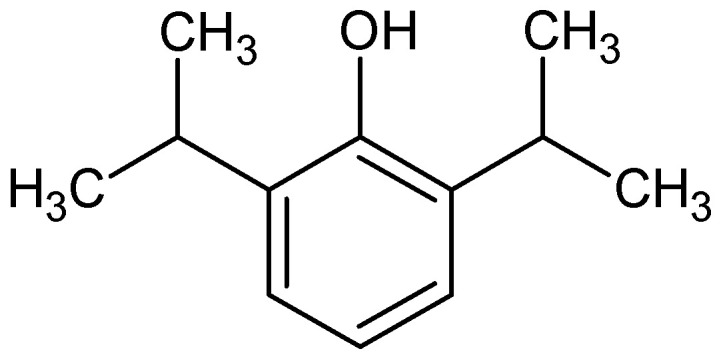
Chemical structure of propofol (**17**).

Propofol, IUPAC name 2,6-diisopropylphenol (Figure 13)

It is a phenol with two isopropyl groups at the 2 and 6 (ortho) positions. It has a melting point of 18 °C, a boiling point of 256 °C, and a log P of 3.79. It is a clear liquid, is slightly soluble in water, and is applied in the form of an emulsion [172]. It is a short-acting intravenous non-barbiturate phenolic anesthetic that has gained widespread use since its introduction in the late 1980s.

Propofol has been demonstrated to exert its effects through multiple mechanisms. Initially, the GABA_A_ receptor is activated, and propofol analogs also function as sodium channel blockers. Some research also suggests that the endocannabinoid system may contribute significantly to the anesthetic action of propofol and to its unique properties [173,174].

It is used in operating rooms for both the induction and maintenance of sedation. It has largely replaced sodium thiopental because recovery from propofol is faster. In addition, its use is relatively inexpensive compared to other anesthetics due to the shorter length of stay in the ICU. Propofol is also used for status epilepticus when other medicines have not been effective. In addition to its favorable anesthetic properties, propofol has a number of non-anesthetic effects. It has anxiolytic properties that may be related to several neurotransmitter systems. It also has antioxidant, immunomodulatory, mild analgesic, antiemetic, and neuroprotective properties. Furthermore, propofol inhibits platelet aggregation and increases intracellular calcium concentration [175,176]. Apart from the anti-inflammatory effect, it also exerts an anti-thrombotic effect by inhibiting the aggregation of blood platelets. It increases nitric oxide production in leukocytes and inhibits thromboxane synthesis in platelets [177].

Propofol binds strongly to proteins in vivo and is metabolized by conjugation in the liver to form glucuronides. Serum albumin and hemoglobin showed marked binding for propofol. A 4% solution of albumin bound 88.7%, and hemoglobin bound 86.2% of the anesthetic [178]. Metabolism in the liver is based on cytochrome P450 isoforms. Low variability was found in the production of the hydroxylated metabolite of propofol, 2,6-diisopropyl-1,4-quinol. [179].

The metabolic pathways of propofol have been studied in several species and are involved in the direct conjugation of the hydroxyl group and aromatic or aliphatic hydroxylation at the 4 (para) position, followed by the conjugation of 2,6-diisopropyl-1,4-quinol with glucuronic acid at the C1 and C4 positions. A small amount of propofol is metabolized at the C4 position by sulfation catalyzed by sulfotransferase. This secondary metabolite is involved in the clearance of propofol in humans. All metabolites are inactive, with the exception of 2,6-diisopropyl-1,4-quinol, which has about a third of the hypnotic activity of propofol [180].

A water-soluble form of the prodrug, fospropofol (a disodium salt of 2,6-diisopropylphenoxymethyl phosphate), was developed and tested. Its pharmacodynamic profile is similar to that of propofol, but the disodium salt of fospropofol has a more rapid onset of action and a longer recovery time because the prodrug must first be converted into the active metabolite. Fospropofol is metabolized in the liver by alkaline phosphatases to propofol, formaldehyde, and phosphate [181,182,183].

**Figure 14:**
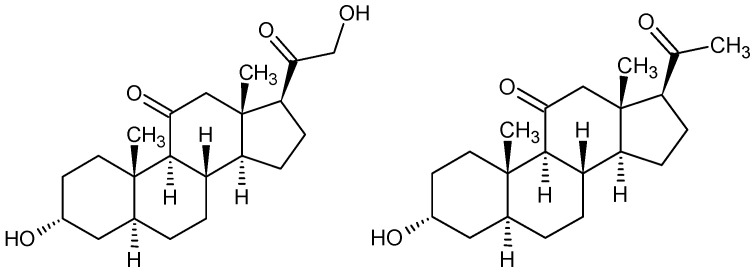
Structures of alfadolone (**18**, left) and alfaxalone (**19**, right).

Steroid anesthetics—a mixture of two steroids, alfadolone (3α,21-dihydroxy-5α-pregnane-11,20-dione), and alfaxalone (3α-hydroxy-5α-pregnane-11,20-dione) (Figure 14)

Chemically, it is a mixture of two synthetic pregnane neurosteroids, alfaxalone, and alfadolone, which are derivatives of progesterone with hydroxy groups [184,185]. The mechanism of action is based on the modulation of GABA_A_ and NMDA receptors [186,187,188]. The neuroactive steroids alfaxalone (9 mg/mL) and alfadolone (3 mg/mL) have been shown to possess a short-acting effect when administered in solution in Cremophor EL, a non-ionic surfactant. This effect has been observed to be therapeutically broad, exhibiting a comparable effect to that of thiopental. Furthermore, thiopental has been demonstrated to be capable of reducing intracranial and intraocular pressure [189,190,191,192,193].

The use of Cremophor^®^ EL as an additive to increase water solubility was found to be the cause of hypersensitivity reactions, leading to its withdrawal from the market. Consequently, the use of sulfobutyl ether-β-cyclodextrin (SBECD) as an excipient to solubilize the drug alfaxolone was studied. The results of this study demonstrated that the anesthetic effect remained unaltered [194], thereby validating its use in veterinary anesthetic practice [195,196,197].

As demonstrated in a study [198], steroid anesthetics contain eight chiral centers, resulting in complex stereochemistry. The effects on GABA_A_ receptor modulation in relation to anesthetic action were evaluated for selected steroids. The results showed that only 5α-steroids exhibited a high degree of stereoselectivity compared to 5β-steroids. These data demonstrate that the recognition site of an anesthetic steroid can discriminate between enantiomers.

Phenylacetic acid derivatives

**Figure 15:**
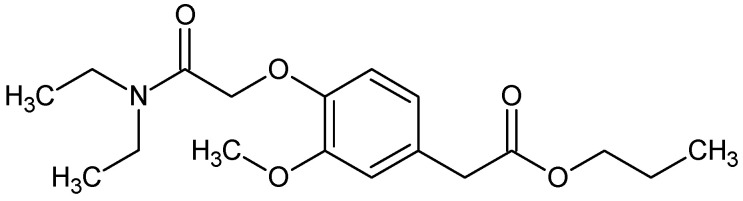
Chemical structure of propanidid (**20**).

Propanidid-IUPAC name: propyl {4-[2-(diethylamino)-2-oxoethoxy]-3-methoxyphenyl}acetate (Figure 15)

Propanidid is a chemical compound that is classified as an ester of phenylacetic acid. It exists as a slightly yellow oil with a boiling point of 210–212 °C. The compound is practically insoluble in water, but it is soluble in alcohol and chloroform [199].

The mechanism of action is based on the activation of γ-aminobutyric acid type A receptors. It was found that the amino acids Asp245, Asp424, Asp425, Arg428, Phe307, and Ser308 played important roles in this receptor binding [200].

Propanidid is an ultra-short-acting phenylacetate-type general anesthetic that was originally introduced by Bayer in 1963. It demonstrated a low incidence of excitatory and respiratory side effects and produced rapid awakening due to its rapid metabolism and a lack of redistribution, in contrast to barbiturates. Its solubility in water was achieved (as a 5% or 20% solution) using polyoxyethylated castor oil (Cremophor EL), which acted as a solubilizing agent. However, Cremophor EL has in some cases caused anaphylactic reactions and elevated histamine levels in humans (both when administered intravenously and orally) [201,202,203]. Subsequent studies have explored the replacement of Cremophor EL with a liposomal preparation, which has demonstrated a higher degree of tolerability [204].

The introduction of alternative, safer, short-acting drugs, such as propofol, resulted in the withdrawal of propanidid from clinical use [205].

## 4. Conclusions

The last 50 years have seen remarkable developments in the field of anesthesia, leading to significant improvements in the efficacy and safety of anesthetics. As a result, a limited but versatile range of anesthetic agents and their adjuvants is available to suit every clinical situation. In contemporary anesthetic practice, isoflurane, sevoflurane, and desflurane are the most commonly used inhalational anesthetics, while propofol is the most prevalent intravenous anesthetic. This state of affairs is widely considered to be a primary factor contributing to the observed stagnation in the development of novel general anesthetics in recent years. Although there have been significant advances in anesthesia over the years, much of this has been achieved through improvements in the administration of anesthesia, such as the availability of specialist anesthetists, advances in the monitoring of anesthesia and the setting of strict standards for anesthesia in clinical practice. Another contributing factor is the use of several anesthetics in combination, as well as the addition of various adjuvants. In the USA, only three general anesthetics were approved between 1985 and 2014, with no new investigational drugs developed for inhalational anesthesia since 1990 [206]. Moreover, most of the new developments in this field are simply modifications of long-established anesthetics. The reasons for this stall in the development are in fact multifaceted. In addition to purely economic factors, pharmacological considerations also play a role. The most significant of these limitations is arguably the current state of understanding regarding the mechanisms of action underlying general anesthesia, since a reliable general theory of the action of anesthetic agents is still not in sight.

In any case, the prevailing level of satisfaction with existing anesthetic agents is not entirely justified. The primary concern pertains to the markedly low safety margins observed, particularly in the context of inhalational anesthetics, which exhibit therapeutic indices ranging from 2 to 4, leading to discernible morbidity and mortality associated with general anesthesia, as well as the necessity for strict control of anesthetic administration. Another issue is postoperative cognitive dysfunction, with potential long-term impairment, especially in elderly patients. Further adverse effects include neurotoxicity in infants and possible interference with cancer therapy. Major issues in intravenous anesthetics are their propensity to central nervous system excitation and problems with solubility [207].

Presently, a significant proportion of the development of new anesthetics is focused on the advancement of intravenous anesthesia [208,209,210]. For instance, fospropofol was developed to enhance solubility and mitigate the incidence of pain during injection. Fospropofol acts as a prodrug, undergoing progressive conversion in vivo to yield propofol and formaldehyde. Other investigational anesthetics are the derivatives of etomidate (e.g., ABP-700), with research aiming to reduce undesired effects, particularly the propensity for adrenocortical depression [209]. Hence, an important issue is the development of anesthetic agents that possess neuroprotective properties [211]. An innovative approach is the loading of anesthetics onto nanocarriers, especially lipid-based nanoparticles [212].

The aforementioned considerations also pertain to the development of novel drugs derived from single enantiomers of existing anesthetics. With regard to the differences in the efficacy and toxicity of the individual enantiomers of the chiral drugs, they can be classified in the following ways: (a) both enantiomers have identical efficacy and toxicity; (b) the enantiomers have the same therapeutic and toxic effects but differ in the magnitude of these effects; (c) only one of the enantiomers is pharmacologically active, while the other does not show any activity.; (d) both enantiomers display pharmacological activity but with qualitatively different therapeutic and toxic effects [213]. All these options also apply for chiral anesthetics, with (b) probably being the most prominent. Qualitatively different (enantiospecific) effects are also plausible, as in the case of the enantiomers of ketamine [214]. The differences in the activity of, e.g., isoflurane enantiomers are much lower. However, despite the generally minor difference in the activities of the individual enantiomers, they can, in principle, indicate a significant improvement in comparison to the utilization of the compound in its racemic form, especially when their low therapeutic indices are taken into account. Even modestly increasing anesthetic efficacy without increasing toxicity could have substantial clinical benefits [215]. It is evident that the overall effects are the result of the interplay of several enantioselective processes, including differential effects on drug distribution, plasma protein binding, metabolism, and elimination. Therefore, the effects are not solely due to their different intrinsic activities at (potential) receptor sites.

Thus, evident advantages exist pertaining to the utilization of enantiomerically pure anesthetic agents. Nevertheless, their employment is constrained, mainly by economic factors. The synthesis of enantiomerically pure isomers of chiral anesthetics can be a challenging task, frequently necessitating a laborious and costly combination of enantioselective synthesis and/or preparative isomer separation methods [213,216,217].

In conclusion, it may be argued that the consideration of the chiral structure of general anesthetics is beneficial, not only in the development of new, more effective and safe general anesthetics, but also in the elucidation of the general mechanism of their action, which is not yet fully understood, and may lead to novel anesthetics based on innovative principles.

## Figures and Tables

**Table 1:** Selected properties of inhalational anesthetics [4].

Anesthetic Chemical Name	Bp [°C]	Octanol–Water Partition Coeff.	Blood–Gas Partition Coeff.	Period of Common Use
N_2_O (**1**) *nitrous oxide*	−90	3.2	0.47	1844 to present
xenon (**2**)	−108.1	19	0.12	1950s to present
cyclopropane (**3**)	−32.9	52.5	0.55	1930 to mid-1980s
diethyl ether (**4**)	34.6	6.8	12.1	1846 to 1960s
halothane (**5**) *2-bromo-2-chloro-1,1,1-trifluoroethane*	50	330	2.4	1956 to 1980s
isoflurane (**6**) *2-chloro-2-(difluoromethoxy)-1,1,1-trifluoroethane*	48.5	174	1.4	1970s to present
enflurane (**7**) *2-chloro-1-(difluoromethoxy)-1,1,2-trifluoroethane*	56.5	120	1.9	1968 to 1990s
desflurane (**8**) *2-(difluoromethoxy)-1,1,1,2-tetrafluoroethane*	22.8	27.2	0.42	1991 to present
sevoflurane (**9**) *1,1,1,3,3,3-hexafluoro-2-(fluoromethoxy)propane*	58.6	56	0.63	1990 to present
methoxyflurane (**10**) *2,2-dichloro-1,1-difluoro-1-methoxyethane*	105	400	12	1960 to late 1970s

**Table 2:** Selected properties of intravenous anesthetics [4].

Anesthetic Chemical Name	pK_a_	Water-Soluble	Melting Point [°C]	Introduced
thiopental (**11**) *5-ethyl-5-pentan-2-yl-2-sulfanylidene-1,3-diazinane-4,6-dione*	7.5	yes	158–160	1934
methohexital (**12**) *5-hex-3-yn-2-yl-1-methyl-5-prop-2-enyl-1,3-diazinane-2,4,6-trione*	7.9	yes	96	1950s
hexobarbital (**13**) *5-(cyclohex-1-en-1-yl)-1,5-dimethylpyrimidine-2,4,6(1H,3H,5H)-trione*	8.2	yes	146.5	1940s
etomidate (**14**) *ethyl 3-[(1R)-1-phenylethyl]imidazole-5-carboxylate*	4.24	no	67	1972
ketamine (**15**) *(RS)-2-(2-chlorophenyl)-2-(methylamino)cyclohexanone*	7.5	yes	92	1970s
esketamine (**16**) *(2S)-2-(2-chlorophenyl)-2-(methylamino)cyclohexan-1-one*	7.5	yes	92	1990s
propofol (**17**) *2,6-diisopropylphenol*	11.0	no	18	1970s
alfadolone (**18**) *3α,21-dihydroxy-5α-pregnane-11,20-dione*	12.9	no	175–177 *	1971
alfaxalone (**19**) *3α-hydroxy-5α-pregnane-11,20-dione*	15.0	no	172	1971
propanidid (**20**) *propyl {4-[2-(diethylamino)-2-oxoethoxy]-3-methoxyphenyl}acetate*	-	no	<25	1963

* as acetate.

**Table 3:** Selected pharmacodynamic and pharmacokinetic properties of common general anesthetics [41].

**Inhalational Anesthetics**					
Anesthetic	MAC *	% metabolized	Volume of distribution ** [L/kg]		Vapor pressure [20 °C, mm Hg]
N_2_O (**1**)	104	0.004			
isoflurane (**6**)	1.17	0.17	4.3		240
desflurane (**8**)	6.6	0.02	0.7		669
sevoflurane (**9**)	1.8	5	1.7		160
**Intravenous anesthetics**					
Anesthetic	Induction dose [mg/kg]	Duration of action [min]	Volume of distribution [L/kg]	Protein binding [%]	Clearance [mL/kg/min]
thiopental (**11**)	3–5	5–10	2.5	83	3.4
etomidate (**14**)	0.2–0.3	3–8	2.5–4.5	77	18–25
ketamine (**15**)	1–2	5–10	3.1	12	12–17
propofol (**17**)	1–2.5	3–8	2–10	97	20–30

* minimum alveolar concentration; ** total volume of distribution at steady state.

## References

[1] Eger E.I., Saidman L.J., Westhorpe R.N. (2014). The Wondrous Story of Anesthesia.

[2] Temple E. (2019). Inhalational anaesthetic agents. Anaesth. Intensive Care Med..

[3] Smith T.C., Smith T., Pinnock C., Lin T., Jones R. (2008). Anaesthetic gases and vapours. Fundamentals of Anaesthesia.

[4] Steihilber D., Schubert-Zsilavecz M., Roth H.J. (2005). Medizinische Chemie. Targets und Arzneistoffe.

[5] Mirsattari S.M., Sharpe M.D., Young G.B. (2004). Treatment of refractory Status Epilepticus with inhalational anesthetic agents isoflurane and desflurane. Arch. Neurol..

[6] Zeiler F.A., Zeiler K.J., Teitelbaum J., Gillman L.M., West M. (2015). Modern inhalational anesthetics for refractory Status Epilepticus. Can. J. Neurol. Sci..

[7] Wolf A., Selpien H., Haberl H., Unterberg M. (2021). Does a combined intravenous-volatile anesthesia offer advantages compared to an intravenous or volatile anesthesia alone: A systematic review and meta-analysis. BMC Anesthesiol..

[8] Radkowski P., Czajka A., Dawidowska-Fidrych J., Braczkowska-Skibińska M. (2024). Past, present and future of intravenous anesthetics. Farm. Pol..

[9] Frauenknecht J., Kirkham K.R., Jacot-Guillarmod A., Albrecht E. (2019). Analgesic impact of intra-operative opioids vs. opioid-free anaesthesia: A systematic review and meta-analysis. Anaesthesia.

[10] Erdoes G., Basciani R.M., Eberle B. (2014). Etomidate—A review of robust evidence for its use in various clinical scenarios. Acta Anaesthesiol. Scand..

[11] Pérez-Bárcena J., Llompart-Pou J.A., Homar J., Abadal J.M., Raurich J.M., Frontera G., Brell M., Ibáñez J., Ibáñez J. (2008). Pentobarbital versus thiopental in the treatment of refractory intracranial hypertension in patients with traumatic brain injury: A randomized controlled trial. Crit. Care.

[12] Chaggar R.S., Campbell J.P. (2017). The future of general anaesthesia in obstetrics. BJA Educ..

[13] Jean-Michel V., Caulier T., Delannoy P.Y., Meybeck A., Georges H. (2022). Thiopental as substitute therapy for critically ill patients with COVID-19 requiring mechanical ventilation and prolonged sedation. Med. Intensiv..

[14] Sheikh S., Hendry P. (2018). The expanding role of ketamine in the emergency department. Drugs.

[15] Lambert D.G. (2020). Mechanism of action of general anesthetic drugs. Anaesth. Intensive Care Med..

[16] Son Y. (2010). Molecular mechanisms of general anesthesia. Korean J. Anesthesiol..

[17] Diao S., Ni J., Shi X., Liu P., Xia W. (2014). Mechanisms of action of general anesthetics. Front. Biosci..

[18] Hemmings H.C., Riegelhaupt P.M., Kelz M.B., Solt K., Eckenhoff R.G., Orser B.A., Goldstein P.A. (2019). Towards a comprehensive understanding of anesthetic mechanisms of action: A decade of discovery. Trends Pharmacol. Sci..

[19] Pavel M.A., Petersen E.N., Wang H., Lerner R.A., Hansen S.B. (2020). Studies on the mechanism of general anesthesia. Proc. Natl. Acad. Sci. USA.

[20] Durga P., Singham G., Baradaa A. (2023). Understanding the GABAA receptor: Implications for anesthesia and beyond. Neuroanaesthesiol. Crit. Care.

[21] Petrenko A.B., Yamakura T., Sakimura K., Baba H. (2014). Defining the role of NMDA receptors in anesthesia: Are we there yet?. Eur. J. Pharmacol..

[22] Zhang Y., Laster M.J., Hara K., Harris R.A., Eger E.I., Stabernack C.R., Sonner J.M. (2003). Glycine receptors may mediate part of the immobility produced by inhaled anesthetics. Anesth. Analg..

[23] Martin D.C., Plagenhoef M., Abraham J., Dennison R.L., Aronstam R.S. (1995). Volatile anesthetics and glutamate activation of N-methyl-d-aspartate receptors. Biochem. Pharmacol..

[24] Dringenberg H.C. (2000). Serotonergic receptor antagonists alter responses to general anaesthetics in rats. Br. J. Anaesth..

[25] Weir C.J., Mitchell S.J., Lambert J.J. (2017). Role of GABA A receptor subtypes in the behavioural effects of intravenous general anaesthetics. Br. J. Anaesth..

[26] Cordato D.J., Chebib M., Mather L.E., Herkes G.K., Johnston G.A. (1999). Stereoselective interaction of thiopentone enantiomers with the GABA(A) receptor. Br. J. Pharmacol..

[27] Dickinson R., de Sousa S.L., Lieb W.R., Franks N.P. (2002). Selective synaptic actions of thiopental and its enantiomers. Anesthesiology.

[28] Coates K.M., Mather L.E., Johnson R., Flood P. (2001). Thiopental is a competitive inhibitor at the human alpha7 nicotinic acetylcholine receptor. Anesth. Analg..

[29] Downie D.L., Franks N.P., Lieb W.R. (2000). Effects of thiopental and its optical isomers on nicotinic acetylcholine receptors. Anesthesiology.

[30] Yagmurdur H., Ayyildiz A., Karaguzel E., Ogus E., Surer H., Caydere M., Nuhoglu B.C., Germiyanoglu C. (2006). The preventive effects of thiopental and propofol on testicular ischemia-reperfusion injury. Acta Anaesth. Scand..

[31] Varughese S., Ahmed R. (2021). Environmental and occupational considerations of anesthesia: A narrative review and update. Anesth. Analg..

[32] Corbett T.H., Cornell R.G., Endres J.L., Lieding K. (1974). Birth defects among children of nurse-anesthetists. Anesthesiology.

[33] Acharya N.K., Goldwaser E.L., Forsberg M.M., Godsey G.A., Johnson C.A., Sarkar A., DeMarshall C., Kosciuk M.C., Dash J.M., Hale C.P. (2015). Sevoflurane and isoflurane induce structural changes in brain vascular endothelial cells and increase blood-brain barrier permeability: Possible link to postoperative delirium and cognitive decline. Brain Res..

[34] Mishra L.D., Agarwal A., Singh A.K., Sriganesh K. (2024). Paving the way to environment-friendly greener anesthesia. J. Anaesthesiol. Clin. Pharmacol..

[35] Khalil R., Ma Z., Lubarsky D., Peng K., Ji F., Liu H. (2024). The environmental effects of anesthetic agents and anesthesia practices. J. Anesth. Transl. Med..

[36] Langbein T., Sonntag H., Trapp D., Hoffmann A., Malms W., Röth E.P., Mörs V., Zellner R. (1999). Volatile anaesthetics and the atmosphere: Atmospheric lifetimes and atmospheric effects of halothane, enflurane, isoflurane, desflurane and sevoflurane. Br. J. Anaesth..

[37] Gaya da Costa M., Kalmar A.F., Struys M.M.R.F. (2021). Inhaled anesthetics: Environmental role, occupational risk, and clinical use. J. Clin. Med..

[38] Hanna M., Bryson G.L. (2019). A long way to go: Minimizing the carbon footprint from anesthetic gases. Can. J. Anesth..

[39] Ravishankara A.R., Daniel J.S., Portmann R.W. (2009). Nitrous oxide (N_2_O): The dominant ozone-depleting substance emitted in the 21st century. Science.

[40] Yasny J.S., White J. (2012). Environmental implications of anesthetic gases. Anesth. Prog..

[41] Pardo M.S. (2023). Miller’s Basics of Anesthesia.

[42] Crawford J., Lewis M. (1986). Nitrous oxide in early human pregnancy. Anaesthesia.

[43] Mazze R.I., Fujinaga M., Rice S.A., Harris S.B., Baden J.M. (1986). Reproductive and teratogenic effects of nitrous oxide, halothane, isoflurane, and enflurane in Sprague-Dawley rats. Anesthesiology.

[44] Gage A.A., Baust J.G. (2007). Cryosurgery for tumors. J. Am. Coll. Surg..

[45] Franks N.P., Dickinson R., de Sousa S.L., Hall A.C., Lieb W.R. (1998). How does xenon produce anaesthesia (letter). Nature.

[46] Lynch C., Baum J., Tenbrinck R., Weiskopf R.B. (2000). Xenon anaesthesia. Anesthesiology.

[47] Bokoch M.P., Gelb A.W. (2014). From the journal archives: Cyclopropane: Induction and recovery with a bang!. Can. J. Anaesth..

[48] MacDonald A.G. (1994). A short history of fires and explosions caused by anaesthetic agents. Br. J. Anaesth..

[49] Bovill J.G. (2008). Inhalation anaesthesia: From diethyl ether to xenon. Handb. Exp. Pharmacol..

[50] Chang C.Y., Goldstein E., Agarwal N., Swan K.G. (2015). Ether in the developing world: Rethinking an abandoned agent. BMC Anesthesiol..

[51] Conway C.M. (1965). The anaesthetic ethers. Br. J. Anaesth..

[52] Huang L., Sang C.N., Desai M.S. (2017). Beyond ether and chloroform—A major breakthrough with halothane. J. Anesth. Hist..

[53] Gál B., Bucher C., Burns N.Z. (2016). Chiral alkyl halides: Underexplored motifs in medicine. Mar. Drugs.

[54] Egan T. (1996). Stereochemistry and anesthetic pharmacology: Joining hands with the medicinal chemists. Anesth. Analg..

[55] Sedensky M.M., Cascorbi H.F., Meinwald J., Radford P., Morgan P.G. (1994). Genetic differences affecting the potency of stereoisomers of halothane. Proc. Nat. Acad. Sci. USA.

[56] Martin J.L., Meinwald J., Radford P., Liu Z., Graf M.L., Pohl L.R. (1995). Stereoselective metabolism of halothane enantiomers to trifluoroacetylated liver proteins. Drug Metab. Rev..

[57] Mather L.E., Fryirs B.L., Duke C.C., Cousins M.J. (2000). Lack of whole body pharmacokinetic differences of halothane enantiomers in the rat. Anesthesiology.

[58] Mizobe T. (2019). The halothane hepatitis that was not. Br. J. Anaesth..

[59] Bradshaw J.J., Ivanetich K.M. (1984). Isoflurane: A comparison of its metabolism by human and rat hepatic cytochrome P-450. Anesth. Analg..

[60] Kharasch E.D., Hankins D.C., Cox K. (1999). Clinical isoflurane metabolism by cytochrome P450 2E1. Anesthesiology.

[61] Karpinskii T.M., Szulc R., Szyfter K. (2006). Role of cytochrome P450 in metabolism of inhalation anaesthetics. Nov. Lek..

[62] Lysko G.S., Robinson J.L., Casto R., Ferrone R.A. (1994). The stereospecific effects of isoflurane isomers in vivo. Eur. J. Pharmacol..

[63] Franks N.P., Lieb W.R. (1991). Stereospecific effects of inhalational general anesthetic optical isomers on nerve ion channels. Science.

[64] Dickinson R., Franks N.P., Lieb W.R. (1994). Can the stereoselective effects of the anesthetic isoflurane be accounted for by lipid solubility?. Biophys. J..

[65] Moody E.J., Harris B.D., Skolnick P. (1993). Stereospecific actions of the inhalation anesthetic isoflurane at the GABAA receptor complex. Brain Res..

[66] Hall A.C., Lieb W.R., Franks N.P. (1994). Stereoselective and non-stereoselective actions of isoflurane on the GABAA receptor. Br. J. Pharmacol..

[67] Quinlan J.J., Firestone S., Firestone L.L. (1995). Isoflurane’s enhancement of chloride flux through rat brain gammaaminobutyric acid type A receptors is stereoselective. Anesthesiology.

[68] Oz M., Tchugunova Y., Dinc M., Dunn S.M. (2002). Effects of isoflurane on voltage-dependent calcium fluxes in rabbit T-tubule membranes: Comparison with alcohols. Arch. Biochem. Biophys..

[69] Xu Y., Tang P., Firestone L., Zhang T.T. (1996). 19F nuclear magnetic resonance investigation of stereoselective binding of isoflurane to bovine serum albumin. Biophys. J..

[70] Harris B., Moody E., Skolnick P. (1992). Isoflurane anesthesia is stereoselective. Eur. J. Pharmacol..

[71] Eger E.I., Koblin D.D., Laster M.J., Schurig V., Juza M., Ionescu P., Gong D. (1997). Minimum alveolar anesthetic concentration values for the enantiomers of isoflurane differ minimally. Anesth. Analg..

[72] Krasowski M.D., Harrison N.L. (2000). The actions of ether, alcohol and alkane general anaesthetics on GABAA and glycine receptors and the effects of TM2 and TM3 mutations. Br. J. Pharmacol..

[73] Garton K.J., Yuen P., Meinwald J., Thummel K.E., Kharasch E.D. (1995). Stereoselective metabolism of enflurane by human liver cytochrome P450 2E1. Drug Metab. Dispos..

[74] Patel S.S., Goa K.L. (1995). Desflurane. A review of its pharmacodynamic and pharmacokinetic properties and its efficacy in general anaesthesia. Drugs.

[75] Jakobsson J. (2012). Desflurane: A clinical update of a third-generation inhaled anaesthetic. Acta Anaesthesiol. Scand..

[76] Saros G.B., Doolke A., Anderson R.E., Jakobsson J.G. (2006). Desflurane vs. sevoflurane as the main inhaled anaesthetic for spontaneous breathing via a laryngeal mask for varicose vein day surgery: A prospective randomized study. Acta Anaesthesiol. Scand..

[77] Gupta P., Rath G.P., Prabhakar H., Bithal P.K. (2015). Comparison between sevoflurane and desflurane on emergence and recovery characteristics of children undergoing surgery for spinal dysraphism. Indian J. Anaesth..

[78] Dayan A.D. (2016). Analgesic use of inhaled methoxyflurane: Evaluation of its potential nephrotoxicity. Hum. Exp. Toxicol..

[79] Blair H.A., Frampton J.E. (2016). Methoxyflurane: A review in trauma pain. Clin. Drug Investig..

[80] Brioni J.D., Varughese S., Ahmed R., Bein B. (2017). A clinical review of inhalation anesthesia with sevoflurane: From early research to emerging topics. J. Anesth..

[81] Sami A., Burcu A. (2023). Overview of sevoflurane as an volatile anesthetic. Int. J. Clin. Anesthesiol..

[82] Delgado-Herrera L., Ostroff R.D., Rogers S.A. (2001). Sevoflurane: Approaching the ideal inhalational anesthetic. A pharmacologic, pharmacoeconomic, and clinical review. CNS Drug Rev..

[83] Sakai E.M., Connolly L.A., Klauck J.A. (2005). Inhalation anesthesiology and volatile liquid anesthetics: Focus on isoflurane, desflurane, and sevoflurane. Pharmacotherapy.

[84] Patel S.S., Goa K.L. (1996). Sevoflurane. A review of its pharmacodynamic and pharmacokinetic properties and its clinical use in general anaesthesia. Drugs.

[85] Ferrando C., Aguilar G., Piqueras L., Soro M., Moreno J., Belda F.J. (2013). Sevoflurane, but not propofol, reduces the lung inflammatory response and improves oxygenation in an acute respiratory distress syndrome model. Eur. J. Anaesthesiol..

[86] Liu X., Liu X., Xu Y., Xu Z., Huang Y., Chen S., Li S., Liu D. (2020). Ventilatory ratio in hypercapnic mechanically ventilated patients with COVID-19 associated ARDS. Am. J. Respir. Crit. Care Med..

[87] Ode K. (2018). Intravenous anaesthetic agents. Anaesth. Intensive Care Med..

[88] National Center for Biotechnology Information (2024). PubChem Compound Summary for CID 3000715, Thiopental. https://pubchem.ncbi.nlm.nih.gov/compound/Thiopental.

[89] Khan K.S., Hayes I., Buggy D.J. (2014). Pharmacology of anaesthetic agents I: Intravenous anaesthetic agents. Cont. Educ. Anaesth. Crit. Care. Pain..

[90] Nemergut G., Abd-Elsayed A. (2024). Thiopental. Basic Anesthesia Review.

[91] Mather L.E., Edwards S.R. (1998). Chirality in anaesthesia—Ropivacaine, ketamine and thiopentone. Curr. Opin. Anaesthesiol..

[92] Burke D., Henderson D.J. (2002). Chirality: A blueprint for the future. Br. J. Anaesth..

[93] Cordato D.J., Gross A.S., Herkes G.K., Mather L.E. (1997). Pharmacokinetics of thiopentone enantiomers following intravenous injection or prolonged infusion of rac-thiopentone. Br. J. Clin. Pharmacol..

[94] Cordato D.J., Mather L.E., Gross A.S., Herkes G.K. (1999). Pharmacokinetics of thiopental enantiomers during and following prolonged high-dose therapy. Anesthesiology.

[95] Haley T.J., Gidley J.T. (1976). Pharmacological comparison of R(+), S(−) and racemic thiopentone in mice. Eur. J. Pharmacol..

[96] Russo H., Bressolle F. (1998). Pharmacodynamics and pharmacokinetics of thiopental. Clin. Pharmacokinet..

[97] Nguyen K.T., Stephens D.P., McLeish M.J., Crankshaw D.P., Morgan D.J. (1996). Pharmacokinetics of thiopental and pentobarbital enantiomers after intravenous administration of racemic thiopental. Anesth. Analg..

[98] Mather L.E., Edwards S.R., Duke C.C., Cousins M.J. (1999). Enantioselectivity of thiopental distribution into the central neural tissue of rats: An interaction with halothane. Anesth. Analg..

[99] Winters W.D., Spector E., Wallach D.P., Shideman F.E. (1955). Metabolism of thiopental-S35 and thiopental-2-C14 by a rat liver mince and identification of pentobarbital as a major metabolite. J. Pharmacol. Exp. Ther..

[100] Palmer K.M., Fowler M.S., Wall M.E., Rhodes L.S., Waddell W.J., Baggett B. (1969). The metabolism of R(+)- and RS-pentobarbital. J. Pharmacol. Exp. Ther..

[101] Palmer K.H., Fowler M.S., Wall M.E. (1970). Metabolism of optically active barbiturates. II. S-(-)-pentobarbital. J. Pharmacol. Exp. Ther..

[102] Martone C.H., Nagelhout J., Wolf S.M. (1991). Methohexital: A practical review for outpatient dental anesthesia. Anesth. Prog..

[103] Ferguson K., Soin A., Abd-Elsayed A. (2024). Barbiturates. Basic Anesthesia Review.

[104] National Center for Biotechnology Information (2024). PubChem Compound Summary for CID 9034, Methohexital. https://pubchem.ncbi.nlm.nih.gov/compound/Methohexital.

[105] 151-83-7. (Methohexital) ChemicalBook. https://www.chemicalbook.com/ProductChemicalPropertiesCB1492534_EN.htm.

[106] Schwartz R.D., Jackson J.A., Weigert D., Skolnick P., Paul S.M. (1985). Characterization of barbiturate-stimulated chloride efflux from rat brain synaptoneurosomes. J. Neurosci..

[107] Allan A.M., Harris R.A. (1986). Anesthetic and convulsant barbiturates alter gamma-aminobutyric acid-stimulated chloride flux across brain membranes. J. Pharmacol. Exp. Ther..

[108] Luo Y., Balle T. (2022). GABA_A_ receptors as targets for anaesthetics and analgesics and promising candidates to help treat coronavirus infections: A mini-review. Basic Clin. Pharmacol. Toxicol..

[109] Andrews P.R., Mark L.C. (1982). Structural specificity of barbiturates and related drugs. Anesthesiology.

[110] Calvey T.N., Williams N.E. (1997). Intravenous anaesthetics. Principles and Practice of Pharmacology for Anaesthesist.

[111] Welles J.S., McMahon R.E., Doran W.J. (1963). The metabolism and excretion of methohexital in the rat and dog. J. Pharmacol. Exp. Ther..

[112] National Center for Biotechnology Information (2024). PubChem Compound Summary for CID 3608, Hexobarbital. https://pubchem.ncbi.nlm.nih.gov/compound/Hexobarbital.

[113] Knox C., Wilson M., Klinger C.M., Franklin M., Oler E., Wilson A., Pon A., Cox J., Chin N.E., Strawbridge S.A. (2024). DrugBank 6.0: The DrugBank Knowledgebase for 2024. Nucleic Acids Res..

[114] Tseilikman V.E., Kozochkin D.A., Manukhina E.B., Downey H.F., Tseilikman O.B., Misharina M.E., Nikitina A.A., Komelkova M.V., Lapshin M.S., Kondashevskaya M.V. (2016). Duration of hexobarbital-induced sleep and monoamine oxidase activities in rat brain: Focus on the behavioral activity and on the free-radical oxidation. Gen. Physiol. Biophys..

[115] Wahlstrom G. (1966). Differences in anaesthetic properties between the optical antipodes of hexobarbital in the rat. Life Sci..

[116] Takenoshita R., Toki S. (2004). New aspects of hexobarbital metabolism: Stereoselective metabolism, new metabolic pathway via GSH conjugation, and 3-hydroxyhexobarbital dehydrogenases. Yakugaku Zasshi..

[117] Saito K., Dan H., Masuda K., Katsu T., Hanioka N., Yamamoto S., Miyano K., Yamano S., Narimatsu S. (2007). Stereoselective hexobarbital 3′-hydroxylation by CYP2C19 expressed in yeast cells and the roles of amino acid residues at positions 300 and 476. Chirality.

[118] Etomidate. https://www.chemicalbook.com/ChemicalProductProperty_EN_CB4113298.htm.

[119] Godefroi E.F., van der Eijcken C.A.M. (1967). Imidazole Carboxylates. U.S. Patent.

[120] Godefroi E.F., Janssen P.A.J., Van der Eycken C.A.M., Van Heertum A.H.M.T., Niemegeers C.J.E. (1965). DL-(1-arylalkyl)imidazole-5-carboxylate esters: A novel type of hypnotic agents. J. Med. Chem..

[121] Morgan M., Lumley J., Whitwam J.G. (1975). Etomidate, a new water-soluble non-barbiturate intravenous induction agent. Lancet.

[122] Giese J.L., Stanley T.H. (1983). Etomidate: A new intravenous anesthetic induction agent. Pharmacotherapy.

[123] Forman S.A. (2011). Clinical and molecular pharmacology of etomidate. Anesthesiology.

[124] Al Ali M.S., Musa A., Hamadeh W., Seddik E. (2020). Etomidate shows prospect as an anti-arrhythmic drug conferring safe sedation and sinus conversion simultaneously. Dubai Med. J..

[125] Valk B.I., Struys M.M.R.F. (2021). Etomidate and its analogs: A review of pharmacokinetics and pharmacodynamics. Clin. Pharmacokinet..

[126] Tomlin S.L., Jenkins A., Lieb W.R., Franks N.P. (1998). Stereoselective effects of etomidate optical isomers on γ-aminobutyric acid type A receptors and animals. Anesthesiology.

[127] Janssen P.A., Niemegeers C.J., Schellekens K.H., Lenaerts F.M. (1971). Etomidate, R -(+)-ethyl-1-(-methyl-benzyl)imidazole-5-carboxylate (R 16659), a potent, short-acting and relatively atoxic intravenous hypnotic agent in rats. Arzneimittelforschung.

[128] Kaneda K., Yamashita S., Woo S., Han T.H. (2011). Population pharmacokinetics and pharmacodynamics of brief etomidate infusion in healthy volunteers. J. Clin. Pharmacol..

[129] Carlos R., Calvo R., Erill S. (1979). Plasma protein binding of etomidate in patients with renal failure or hepatic cirrhosis. Clin. Pharmacokinet..

[130] Heykants J.J.P., Brugmans J., Doenicke A. On the pharmacokinetics of etomidate (R26490) in human volunteers: Plasma levels, metabolism, and excretion. Clinical Research Report R26490/1 Janssen Research Product Information Service.

[131] Critical Review of Ketamine—WHO Critical Review Report. https://www.scribd.com/doc/77236539/34th-ECDD-2006-Critical-Review-of-Ketamine.

[132] Morgan C.J.A., Curran H.V. (2011). Ketamine use: A review. Addiction.

[133] Hakey P., Ouellette W., Zubieta J., Korter T. (2008). (S)-(+)-Ketamine hydrochloride. Acta Cryst..

[134] O’Neil M.J. (2013). The Merck Index—An Encyclopedia of Chemicals, Drugs, and Biologicals.

[135] Tang Y., Liu R., Zhao P. (2017). Ketamine: An update for obstetric anesthesia. Transl. Perioper. Pain Med..

[136] Barrett W., Buxhoeveden M., Dhillon S. (2020). Ketamine: A versatile tool for anesthesia and analgesia. Curr. Opin. Anaesthesiol..

[137] Arnbjerg J. (1979). Clinical use of ketamine-xylazine for anaesthesia in the cat. Nord. Vet. Med..

[138] Schmidt-Oechtering G.U., Alef M., Röcken M. (1990). Anesthesia of horses with xylazine and ketamine. 2. Anesthesia in adult horses. Tierarzt. Prax..

[139] Lin H.C., Passler T., Wilborn R.R., Taintor J.S., Caldwell F.J. (2015). A review of the general pharmacology of ketamine and its clinical use for injectable anaesthesia in horses. Equine Vet. Educ..

[140] Ibrahim A. (2017). Evaluation of total intravenous anesthesia by ketamine-xylazine constant rate infusion in dogs: A novel preliminary dose study. Vet. Med. Open J..

[141] Baumgartner C., Bollerhey M., Ebner J., Laacke-Singer L., Schuster T., Erhardt W. (2010). Effects of ketamine-xylazine intravenous bolus injection on cardiovascular function in rabbits. Can. J. Vet. Res..

[142] Abdollahpour A., Saffarieh E., Zoroufchi B.H. (2020). A review on the recent application of ketamine in management of anesthesia, pain, and health care. J. Family Med. Prim. Care.

[143] Hana Z., Abdulla S., Alam A., Ma D. (2018). Ketamine: Old drug but new use for neuropathic pain. Transl. Perioper. Pain Med..

[144] Vadivelu N., Schermer E., Kodumudi V., Belani K., Urman R.D., Kaye A.D. (2016). Role of ketamine for analgesia in adults and children. J. Anaesthesiol. Clin. Pharmacol..

[145] Pourmand A., Mazer-Amirshahi M., Royall C., Alhawas R., Shesser R. (2017). Low dose ketamine use in the emergency department, a new direction in pain management. Am. J. Emerg. Med..

[146] Niesters M., Martini C., Dahan A. (2014). Ketamine for chronic pain: Risks and benefits. Br. J. Clin. Pharmacol..

[147] Culp C., Kim H.K., Abdi S. (2021). Ketamine use for cancer and chronic pain management. Front. Pharmacol..

[148] Hashimoto K. (2016). Ketamine’s antidepressant action: Beyond NMDA receptor inhibition. Expert Opin. Ther. Targets.

[149] Berman R.M., Cappiello A., Anand A., Oren D.A., Heninger G.R., Charney D.S., Krystal J.H. (2000). Antidepressant effects of ketamine in depressed patients. Biol. Psychiatry.

[150] Marcantoni W.S., Akoumba B.S., Wassef M., Mayrand J., Lai H., Devantoy S.R., Beauchamp S. (2020). A systematic review and meta-analysis of the efficacy of intravenous ketamine infusion for treatment resistant depression: January 2009—January 2019. J. Affect. Disord..

[151] Witt K., Potts J., Hubers A., Grunebaum M.F., Murrough J.W., Loo C., Cipriani A., Hawton K. (2020). Ketamine for suicidal ideation in adults with psychiatric disorders: A systematic review and meta-analysis of treatment trials. Aust. New Zealand J. Psychiatry.

[152] Wilkowska A., Szałach L., Cubala W.J. (2020). Ketamine in bipolar disorder: A review. Neuropsychiatr. Dis. Treat..

[153] Das R.K., Gale G., Walsh K., Hennessy V.E., Iskandar G., Mordecai L.A., Brandner B., Kindt M., Curran H.V., Kamboj S.K. (2019). Ketamine can reduce harmful drinking by pharmacologically rewriting drinking memories. Nat. Commun..

[154] Krupitsky E., Burakov A., Romanova T., Dunaevsky I., Strassman R., Grinenko A. (2002). Ketamine psychotherapy for heroin addiction: Immediate effects and two-year follow-up. J. Subst. Abuse Treat..

[155] Niquet J., Baldwin R., Norman K., Suchomelova L., Lumley L., Claude G., Wasterlain C.G. (2017). Simultaneous triple therapy for the treatment of status epilepticus. Neurobiol. Dis..

[156] Rosati A., De Masi S., Guerrini R. (2018). Ketamine for refractory status epilepticus: A systematic review. CNS Drugs.

[157] Okon T. (2007). Ketamine: An introduction for the pain and palliative medicine physician. Pain Physician.

[158] Goldman N., Frankenthaler M., Klepacz L. (2019). The efficacy of ketamine in the palliative care setting: A comprehensive review of the literature. J. Palliat. Med..

[159] Dayton P.G., Stiller R.L., Cook D.R., Perel J.M. (1983). The binding of ketamine to plasma proteins: Emphasis on human plasma. Eur. J. Clin. Pharmacol..

[160] Zhao X., Venkata S.L., Moaddel R., Luckenbaugh D.A., Brutsche N.E., Ibrahim L., Zarate C.A., Mager D.E., Wainer I.W. (2012). Simultaneous population pharmacokinetic modelling of ketamine and three major metabolites in patients with treatment-resistant bipolar depression. Br. J. Clin. Pharmacol..

[161] Zanos P., Moaddel R., Morris P.J., Riggs L.M., Highland J.N., Georgiou P., Pereira E.F.R., Albuquerque E.X., Thomas C.J., Zarate C.A. (2018). Ketamine and ketamine metabolite pharmacology: Insights into therapeutic mechanisms. Pharmacol. Rev..

[162] Kamp J., Jonkman K., van Velzen M., Aarts L., Niesters M., Dahan A., Olofsen E. (2020). Pharmacokinetics of ketamine and its major metabolites norketamine, hydroxynorketamine, and dehydronorketamine: A model-based analysis. Br. J. Anaesth..

[163] Dinis-Oliveira R.J. (2017). Metabolism and metabolomics of ketamine: A toxicological approach. Forensic Sci. Res..

[164] Hijazi Y., Boulieu R. (2002). Contribution of CYP3A4, CYP2B6, and CYP2C9 isoforms to N-demethylation of ketamine in human liver microsomes. Drug Metab. Dispos..

[165] Himmelseher S., Pfenninger E. (1998). The clinical use of S-(+)-ketamine a determination of its place. Anästhesiologie Intensivmed. Notfallmedizin Schmerzther..

[166] Zielmann S., Kazmaier S., Schnüll S., Weyland A. (1997). S-(+)-Ketamin und Kreislauf [S-(+)-Ketamine and circulation]. Anaesthesist.

[167] Ihmsen H., Geisslinger G., Schüttler J. (2001). Stereoselective pharmacokinetics of ketamine: R(−)-ketamine inhibits the elimination of S(+)-ketamine. Clin. Pharmacol. Ther..

[168] Nishimura M., Sato K. (1999). Ketamine stereoselectively inhibits rat dopamine transporter. Neurosci. Lett..

[169] Turner E.H. (2019). Esketamine for treatment-resistant depression: Seven concerns about efficacy and FDA approval. Lancet Psychiat..

[170] Wajs E., Aluisio L., Holder R., Daly E.J., Lane R., Lim P., George J.E., Morrison R.L., Sanacora G., Young A.H. (2020). Esketamine nasal spray plus oral antidepressant in patients with treatment-resistant depression: Assessment of long-term safety in a phase 3, open-label study (SUSTAIN-2). J. Clin. Psychiatry.

[171] Canuso C.M., Ionescu D.F., Li X., Qiu X., Lane R., Turkoz I., Nash A.I., Lopena T.J., Fu D.J. (2021). Esketamine nasal spray for the rapid reduction of depressive symptoms in major depressive disorder with acute suicidal ideation or behavior. J. Clin. Psychopharmacol..

[172] Lewis R.J. (1997). Hawley’s Condensed Chemical Dictionary.

[173] Baker M.T., Naguib M. (2005). Propofol: The challenges of formulation. Anesthesiology.

[174] Trapani G., Altomare C., Liso G., Sanna E., Biggio G. (2000). Propofol in anesthesia. Mechanism of action, structure-activity relationships, and drug delivery. Curr. Med. Chem..

[175] Vasileiou I., Xanthos T., Koudouna E., Perrea D., Klonaris C., Katsargyris A., Papadimitriou L. (2009). Propofol: A review of its non-anaesthetic effects. Eur. J. Pharmacol..

[176] Murphy P.G., Myers D.S., Davies M.J., Webster N.R., Jones J.G. (1992). The antioxidant potential of propofol (2,6-diisopropylphenol). Br. J. Anaesth..

[177] Petros A.J., Bogle R.G., Pearson A.D. (1993). Propofol stimulates nitric oxide release from cultured porcine aortic endothelial cells. Br. J. Pharmacol..

[178] Altmayer P., Buch U., Buch H.P. (1995). Propofol binding to human blood proteins. Drug Research.

[179] Guitton J., Buronfosse T., Desage M., Flinois J.P., Perdrix J.P., Brazier J.L., Beaune P. (1998). Possible involvement of multiple human cytochrome P450 isoforms in the liver metabolism of propofol. Br. J. Anaesth..

[180] Raoof A.A., Van Obbergh L.J., De Ville De Goyet J., Verbeeck R.K. (1996). Extrahepatic glucuronidation of propofol in man: Possible contribution of gut wall and kidney. Eur. J. Clin. Pharmacol..

[181] Fechner J., Ihmsen H., Jeleazcov C., Schüttler J. (2009). Fospropofol disodium, a water-soluble prodrug of the intravenous anesthetic propofol (2,6-diisopropylphenol). Expert. Opin. Investig. Drugs.

[182] Welliver M., Rugari S.M. (2009). New drug, fospropofol disodium: A propofol prodrug. AANA J..

[183] Maas A., Maier C., Iwersen-Bergmann S., Madea B., Hess C. (2017). Simultaneous extraction of propofol and propofol glucuronide from hair followed by validated LC–MS/MS analyses. J. Pharm. Biomed. Anal..

[184] National Center for Biotechnology Information (2024). PubChem Compound Summary for CID 104845, Alfaxalone. https://pubchem.ncbi.nlm.nih.gov/compound/Alfaxalone.

[185] National Center for Biotechnology Information (2024). PubChem Compound Summary for CID 24733, Alfadolone Acetate. https://pubchem.ncbi.nlm.nih.gov/compound/Alfadolone-acetate.

[186] Martinez-Botella G., Ackley M.A., Salituro F.G., Doherty J.J. (2014). Natural and synthetic neuroactive steroid modulators of GABAA and NMDA receptors. Annu. Rep. Med. Chem..

[187] Seljeset S., Laverty D., Smart T.G. (2015). Inhibitory neurosteroids and the GABAA receptor. Adv. Pharmacol..

[188] Alvarez L.D., Pecci A. (2018). Structure and dynamics of neurosteroid binding to the α 1 β 2 γ 2 GABAA receptor. J. Steroid. Biochem. Mol. Biol..

[189] Cornet W.T., Popescu D.T. (1977). Althesin (alphadione, CT 1341) a ‘new’ induction agent for anesthesia. Arch. Chir. Neerl..

[190] Kharasch E.D., Hollmann M.W. (2015). Steroid anesthesia revisited: Again. Anesth. Analg..

[191] Towler C.M., Garrett R.T., Sear J.W. (1982). Althesin infusions for maintenance of anaesthesia. Anaesthesia.

[192] Sear J.W. (1996). Steroid anesthetics: Old compounds, new drugs. J. Clin. Anesth..

[193] Manzella F.M., Covey D.F., Jevtovic-Todorovic V., Todorovic S.M. (2022). Synthetic neuroactive steroids as new sedatives and anaesthetics: Back to the future. J. Neuroendocrinol..

[194] Goodchild C.S., Serrao J.M., Kolosov A., Boyd B.J. (2015). Alphaxalone reformulated: A water-soluble intravenous anesthetic preparation in sulfobutyl-ether-β-cyclodextrin. Anesth. Analg..

[195] Muir W., Lerche P., Wiese A., Nelson L., Pasloske K., Whittem T. (2009). The cardiorespiratory and anesthetic effects of clinical and supraclinical doses of alfaxalone in cats. Vet. Anaesth. Analg..

[196] Tamura J., Ishizuka T., Fukui S., Oyama N., Kawase K., Miyoshi K., Sano T., Pasloske K., Yamashita K. (2015). The pharmacological effects of the anesthetic alfaxalone after intramuscular administration to dogs. J. Vet. Med. Sci..

[197] Whittem T., Pasloskhe K.S., Heit M.V., Ranasinghe M.G. (2008). The pharmacokinetics and pharmacodynamics of alfaxalone in cats after single and multiple intravenous administration of Alfaxan at clinical and supraclinical doses. J. Vet. Pharmacol. Ther..

[198] Covey D.F., Nathan D., Kalkbrenner M., Nilsson K.R., Hu Y., Zorumski C.F., Evers A.S. (2000). Enantioselectivity of pregnanolone-induced gamma-aminobutyric acid(A) receptor modulation and anesthesia. J. Pharmacol. Exp. Ther..

[199] Propanidid. https://www.drugfuture.com/chemdata/Propanidid.html.

[200] Wang S., Liu Q., Li X., Zhao X., Qiu L., Lin J. (2018). Possible binding sites and interactions of propanidid and AZD3043 within the γ-aminobutyric acid type A receptor (GABAAR). J. Biomol. Struct. Dyn..

[201] Ball C., Westhorpe R., Kaye G. (2002). Museum of Anaesthetic History. Anaesth. Intensive Care.

[202] Wyant G.M., Zoerb D.L. (1965). Propanidid—A new non-barbiturate intravenous anaesthetic. Can. Anaesth. Soc. J..

[203] Christmas D. (1984). Immune reaction to propanidid. Anaesthesia.

[204] Klockgether-Radke A., Kersten J., Schröder T., Stafforst D., Kettler D., Hellige G. (1995). Anesthesia with propanidid in a liposomal preparation. An experimental study in swine. Der Anaesthesist.

[205] Deschodt J., Lubrano J.F., Peschaud J.L., Eledjam J.J., du Cailar J. (1988). Comparison of propofol and propanidid administered at a constant rate. Ann. Fr. Anesth. Reanim..

[206] Vlassakov K.V., Kissin I. (2016). Decline in the development of new anesthetics. Trends Pharmacol. Sci..

[207] Sneyd J.R. (2017). Thiopental to desflurane—An anaesthetic journey. Where are we going next?. Br. J. Anaesth..

[208] Sneyd J.R., Rigby-Jones A.E. (2010). New drugs and technologies, intravenous anaesthesia is on the move (again). Br. J. Anaesth..

[209] Vellinga R., Valk B.I., Absalom A.R., Struys M.M.R.F., Barends C.R.M. (2022). What’s new in intravenous anaesthesia? New hypnotics, new models and new applications. J. Clin. Med..

[210] Chitilian H.V., Eckenhoff R.G., Raines D.E. (2013). Anesthetic drug development: Novel drugs and new approaches. Surg. Neurol. Int..

[211] Forman S.A. (2010). Molecular approaches to improving general anesthetics. Anesthesiol. Clin..

[212] Zhang S., Wang Y., Zhang S., Huang C., Ding Q., Xia J., Wu D., Gao W. (2023). Emerging anesthetic nanomedicines: Current state and challenges. Int. J. Nanomedicine.

[213] Aboul-Enein H.Y., Bojarski J., Szymura-Oleksiak J. (2000). The impact of chirality of the fluorinated volatile inhalation anaesthetics on their clinical applications. Biomed. Chromatogr..

[214] Calvey T.N. (1995). Isomerism and anaesthetic drugs. Acta Anaesthesiol. Scand. Suppl..

[215] Moody E.J., Harris B., Hoehner P., Skolnick P. (1994). Inhibition of [3H] isradipine binding to L-type calcium channels by the optical isomers of isoflurane. Lack of stereospecificity. Anesthesiology.

[216] Huang C.G., Rozov L.A., Halpern D.F., Vernice G.G. (1993). Preparation of the isoflurane enantiomers. J. Org. Chem..

[217] Juza M., Braun E., Schurig V. (1997). Preparative enantiomer separation of the inhalation anesthetics enflurane, isoflurane and desflurane by gas chromatography on a derivatized gamma-cyclodextrin stationary phase. J. Chromatogr. A.

